# Supplementary figures and images for: Principal component analysis of coronaviruses reveals their diversity and seasonal and pandemic potential
Source: PLoS One. 2020 Dec 3;15(12):e0242954. doi: 10.1371/journal.pone.0242954 (PMC7714145; doi:10.1371/journal.pone.0242954)

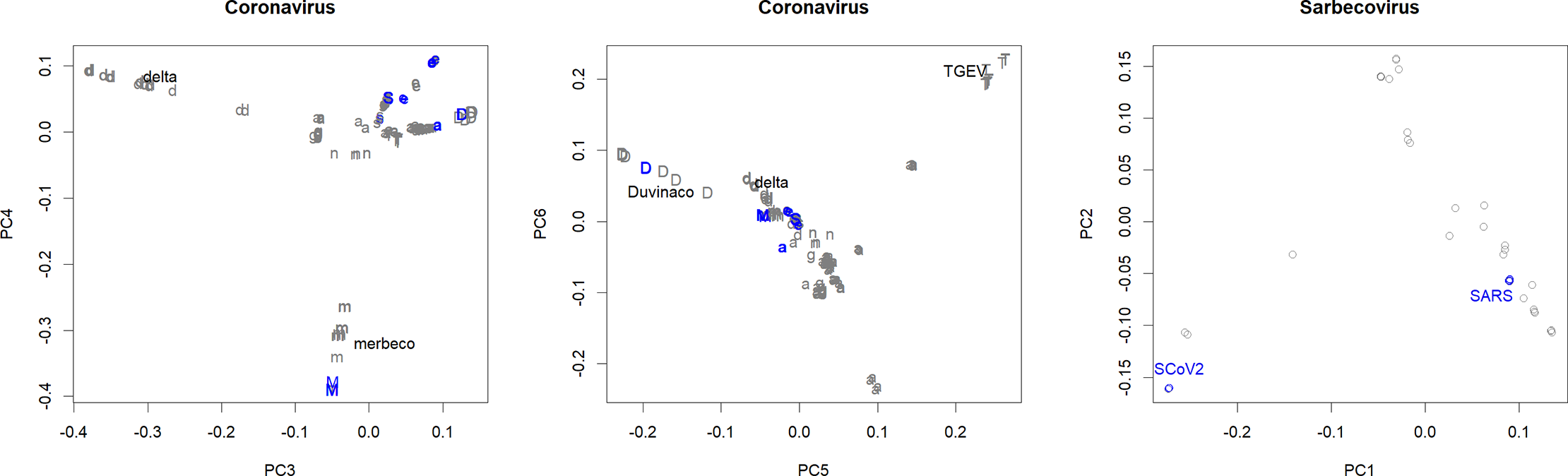

Supplement: S1 Fig — Blue: human samples. Labels are the same as in Fig 1C. Classes found in the Sarbecovirus. SARS-CoV and SCoV2 belong to different groups. (TIF) [file pone.0242954.s001.tif]

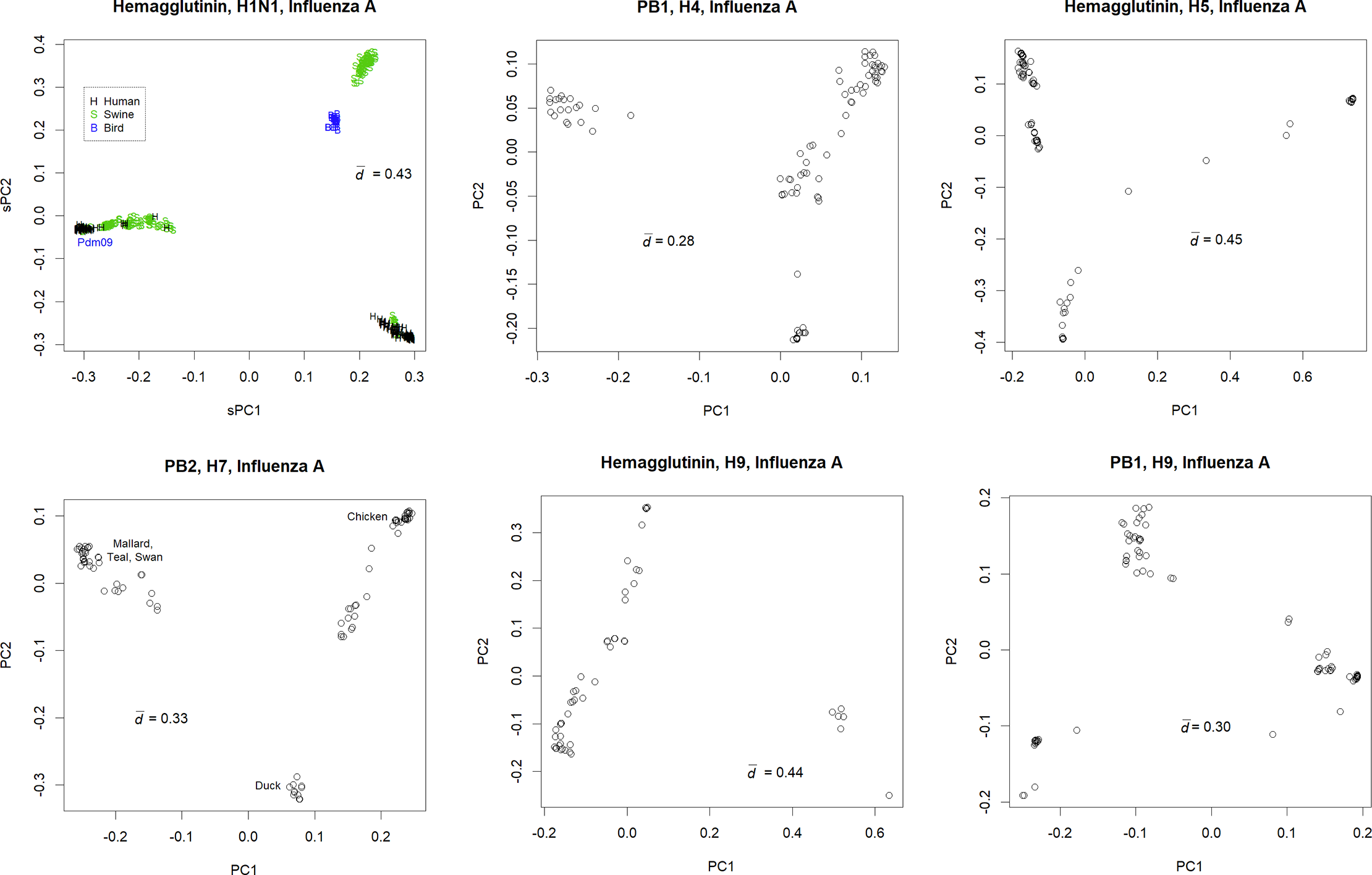

Supplement: S2 Fig — a. H1 hemagglutinin, b. H4 PB1, c. H5 hemagglutinin, d. H7 PB2, e. H9 hemagglutinin, f. H9 PB1. Values of the mean distance were indicated. The subclass may coincide with the hosts (d) but in many cases, one host species formed a distinct class. (TIF) [file pone.0242954.s002.tif]

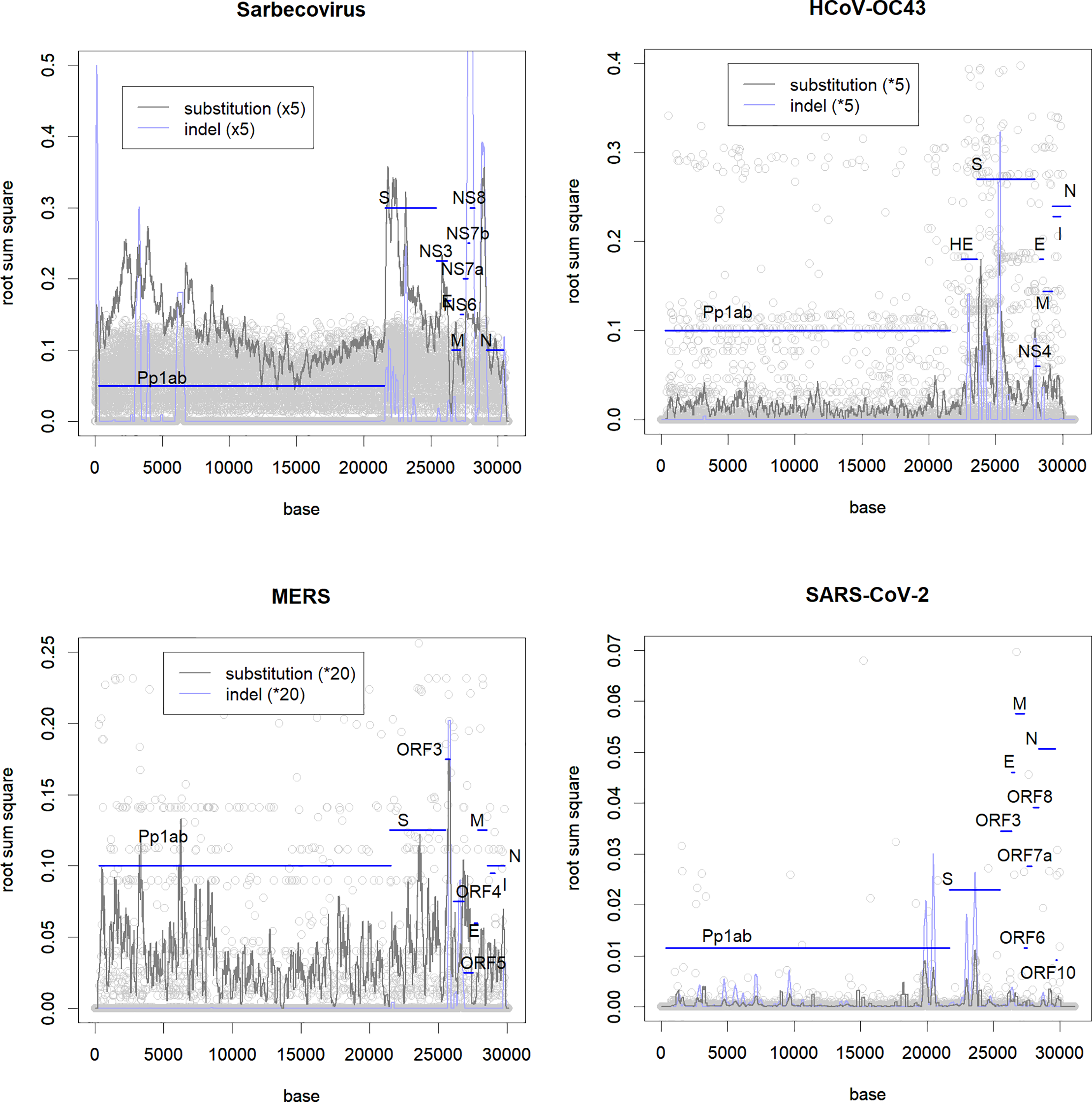

Supplement: S3 Fig — a. Sarbecovirus, b. HCoV OC43, c. MERS-CoV. d. SCoV2. Names of the ORFs are indicated in Fig 2. (TIF) [file pone.0242954.s003.tif]

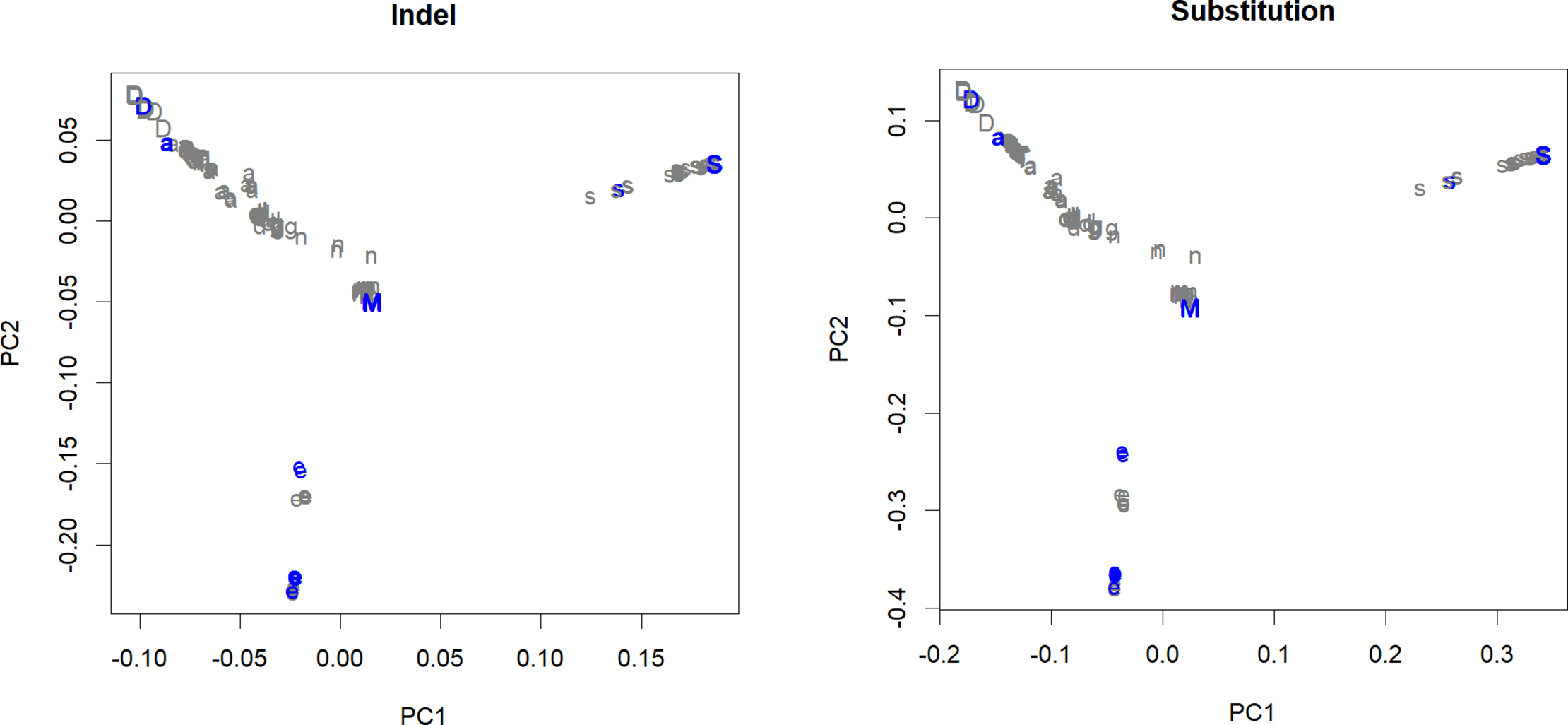

Supplement: S4 Fig — Estimated using indels (a) and substitutions (b). (TIF) [file pone.0242954.s004.tif]

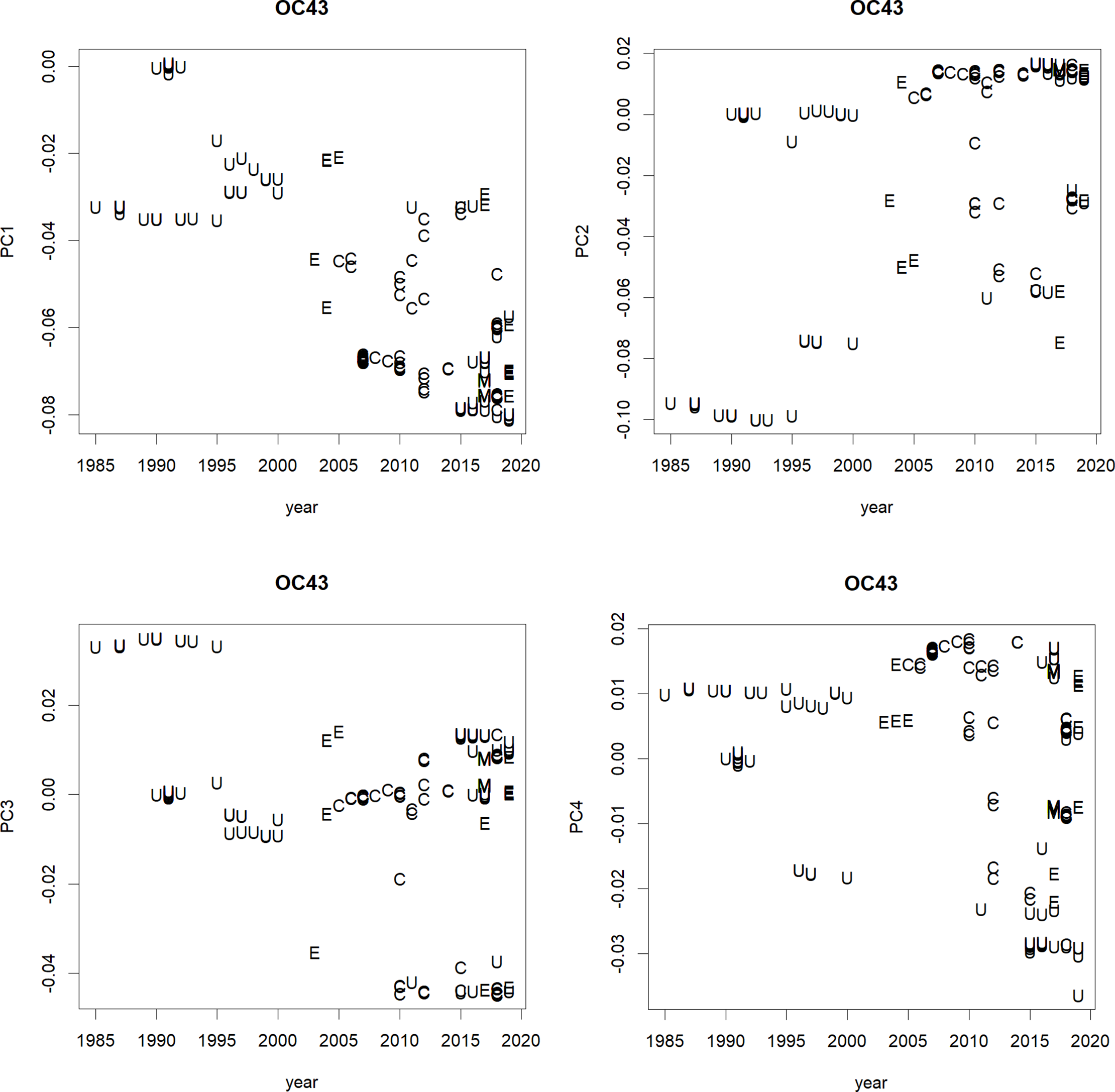

Supplement: S5 Fig — Conservative characteristics of the virus and repeated appearance were obvious. (TIF) [file pone.0242954.s005.tif]

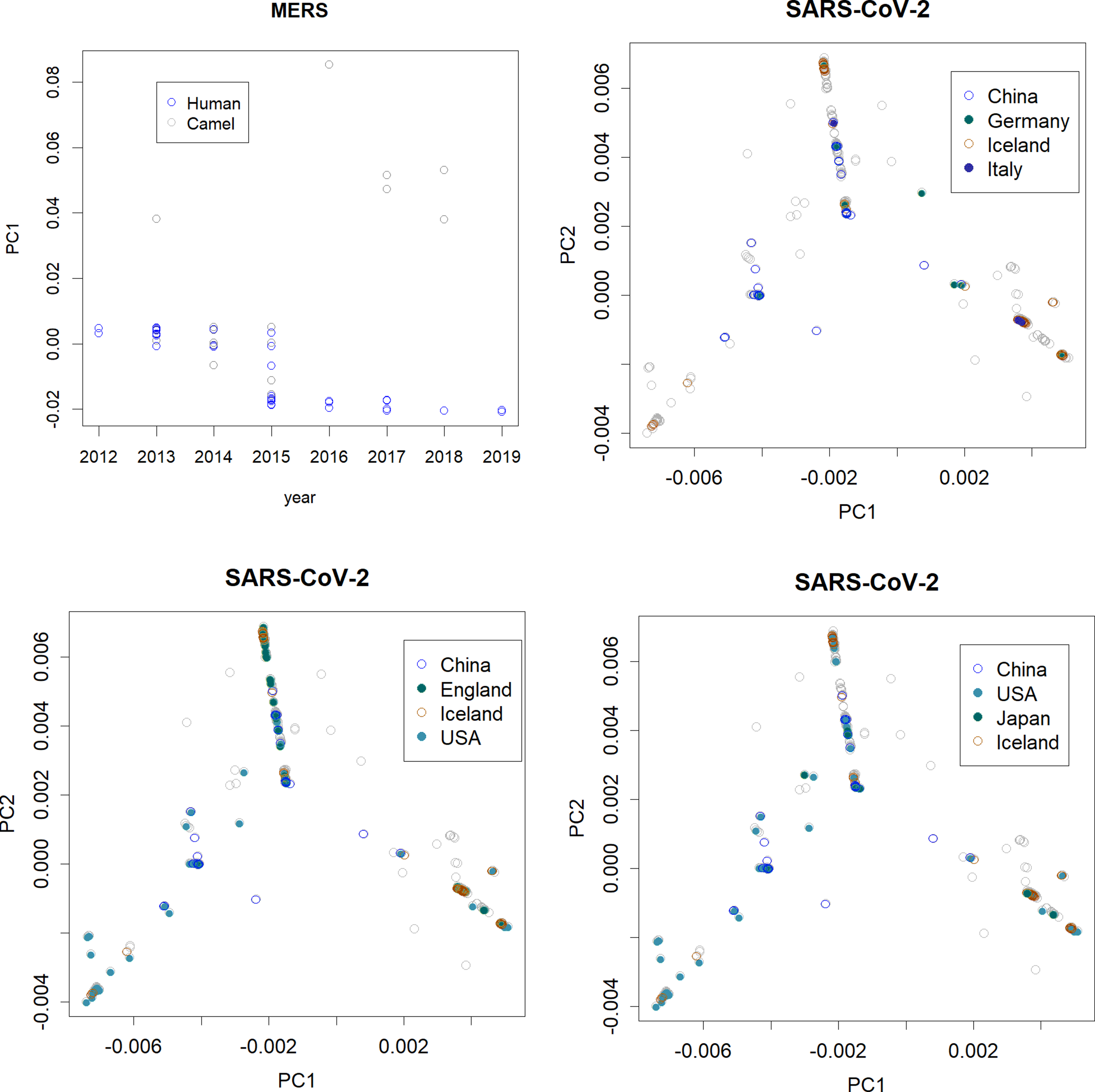

Supplement: S6 Fig — a. Annual changes in the MERS-CoV genome. b. Comprehensive data for SARS-CoV-2. Samples found in some European countries showed higher magnitudes of PCs, indicating accumulation of mutations (also see S4 Table). (TIF) [file pone.0242954.s006.tif]
