# Supplementary material for: Principal component analysis of coronaviruses reveals their diversity and seasonal and pandemic potential
Source: PLoS One. 2020 Dec 3;15(12):e0242954. doi: 10.1371/journal.pone.0242954 (PMC7714145; doi:10.1371/journal.pone.0242954)
Supplement: Attachment — Submitted filename: PONE-D-20-16191_R1_highlighted with comments.pdf [file pone.0242954.s014.pdf]

PLOS ONE

Principal component analysis of coronaviruses reveals their diversity and seasonal and pandemic potential.

--Manuscript Draft--

Manuscript Number:	PONE-D-20-16191R1
Article Type:	Research Article
Full Title:	Principal component analysis of coronaviruses reveals their diversity and seasonal and pandemic potential.
Short Title:	Coronaviruses, a source of pandemic and seasonal pathogens.
Corresponding Author:	Tomokazu Konishi<br>Akita Prefectural University<br>Akita, JAPAN
Keywords:	annual changes of virus; candidates of subsequent pandemics; classification of coronavirus; host specificity; seasonal outbreak
Abstract:	Coronaviruses and influenza viruses have similarities and differences. In order to comprehensively compare them, their genome sequencing data were examined by principal component analysis. Coronaviruses had fewer variations than a subclass of influenza viruses. In addition, differences among coronaviruses that infect a variety of hosts were also small. These characteristics may have facilitated the infection of different hosts. Although many of the coronaviruses were conservative, those repeatedly found among humans showed annual changes. If SARS-CoV-2 changes its genome like the Influenza H type, it will repeatedly spread every few years. In addition, the coronavirus family has many other candidates for new pandemics.
Order of Authors:	Tomokazu Konishi
Response to Reviewers:	>1. Lines 28-29: Please add a reference to this sentence. The meaning is not being understood. How the author concludes that the limited variations in CoVs “eased the novel transfection of CoV to humans” needs to be elaborated a little.<br><br>I deleted Lines 28-29 to satisfy a request by reviewer 2. It was a summary of the article. Instead, the conclusions were drawn at the end of the Discussion. Additionally, I altered the sentence to explain why the new strain will infect humans with ease. I hope that the meaning is now clear.<br><br>>2. Line 37: it must be “nearly 30 kb”.<br><br>I changed the sentence accordingly.<br><br>>3. Lines 37-38: what about influenza genome. It is single stranded RNA but negative sense segmented genome of about 3.5 kb size. The comparative parameters must be same in one sentence for an apt comparison.<br><br>I altered the paragraph and explained the basis of comparisons, adding to the explanation of comparison in the materials and methods.<br><br>>4. Lines 39-40: Add a reference to this statement.<br><br>I added a review article that explains that RNA-dependent RNA polymerase has lower fidelity and how this contributes to the evolution of the virus.<br><br>>5. Lines 43-46: Reference for an “another large study” is also same, i.e. ref. no. 6. Pl. check the appropriate ref.<br><br>I apologize for the wrong citation. I have corrected it.<br><br>Also, here it is important to mention the current infectivity and case fatality rates for human CoVs and influenza viruses.

Fatality rates are still difficult to compare. However, I mentioned this and cited new references.

>6. Lines 55- 56, 109 and others: Full forms for abbreviations are required at places of first mention: DDBJ, DECIPHER, TGEV, etc. throughout the manuscript.

I have added the full forms for DDBJ, TGEV, SARS, MARS, HCoV, and COVID. However, DECIPHER is not an abbreviation but a unique noun given by the developer "DECIPHER is a software toolset that can be used for deciphering and managing biological sequences efficiently using the R programming language."

>7. Materials and Methods: This section needs extensive revision. What were the study samples or groups and their sources have not been indicated? Were sequences of all the three, i.e., SARS-CoV, MERS-CoV and SARS-CoV-2 were analyzed?

All the sequences used are available in the Supporting Information files. As GISAID did not allow us to share the sequences, only the list was given.

>8. Line 57: What kind of "samples"?

I added the term "virus."

>9. Line 64: Were only 2796 full-length data available or this much were narrowed down based on any particular parameters? If the latter, explain those parameters used for short-listing the desired sequences. And why others were excluded.

As was written, all 2796 data points were used in Fig 3C. One of the advantages of PCA is that it is capable of handling a large number of samples. Now, I am submitting a new article, which uses many more virus samples to see the present state of the pandemic.

>10. The methodology section should be made very clear to make it understandable for even a novice in the area. It is especially more important for such an article that addresses the current COVID-19 pandemic.

I added a paragraph explaining PCA. I hope that this gives enough information.

>11. It has been stated that the CoVs mutate less but evolve more (add ref.). Please discuss about this also.

I am afraid there was a misinterpretation. HCoVs are conservative (Fig. 3A) and, with the current data, the evolutionary rate of SARS-CoV-2 cannot be estimated. Coronaviruses have not evolved rapidly when compared with influenza (Fig. 1, S2A Fig.).
HCoVs are more conservative than flu viruses (Fig. 3A). The annual change is very small (Fig. 3B) in comparison with the influenza N1H1. To clarify this, I added a Supporting figure (S2G Fig.) where the difference in the evolutionary rate is very clear.

>12. Line 128: Please revise this sentence for aptness. Human outbreak strains must be replaced with more appropriate scientific terminology, for eg., Human CoVs of epidemic potential or epidemic human CoVs.

I replaced the term accordingly.

>13. Line 129: "SARS spike protein" to be replaced with "spike protein of SARS-CoV".

I replaced the wording accordingly.

>14. The scientific aptness for usage of words needs to be carefully proof-read throughout the manuscript.
Unfortunately, English is not my first language, and so as was stated, the manuscript was professionally edited for language before submission. I have reordered English

editing. I hope that this improves the manuscript.

>15. Lines 135- 136: Can this statement be supported by any other epidemiological study (studies based on respiratory clinical specimens and sero-surveillance) reference?

Unfortunately, no. The differences in the viruses are not detectable by ordinary clinical or serum analysis. Only DNA sequencing can reveal differences. However, for a long time, we used the wrong methods for sequence analysis; this might have obscured many obvious pieces of evidence. I refer to a new figure added in the Supporting Information.

>16. Lines 138-139: Kindly re-frame the sentence for better clarity as this seems one of the important observations of the study. For eg., what "variety" is being referred to here?

I have added explanations to clarify what was selected.
I wish to thank the reviewer for the comment. I found that the European sample (Normandy, France) was reported in 2017, but it was collected in 2002. I corrected Fig 2A and 2B accordingly.

>17. Even though the data is depicted in figures, the text should maintain clarity of thoughts of the authors.

I have reordered the English edition. I hope that this improves the manuscript.

>18. Line 147: contrastive to be replaced by "in contrast".

I replaced the wording accordingly.

>19. The manuscript needs to be extensively revised for usage of English language in order to aptly express the important scientific findings of this work.

I have reordered English editing. I hope that this improves the manuscript.

>20. Line 152: "Emvecovirus" to be corrected "Embecovirus".

I replaced the wording accordingly.

>21. Lines 159-162: Important statement, but requires revision for clarity. For instance, it can be "The spike protein mediated infection by CoVs…more adaptable…"

I replaced the wording accordingly.

>22. Line 176-177: "Adaptation to the genetic system of a new host may alter codon usage and several amino acids" needs reference.

I added a reference.

>23. Line 185: "SCoV2 satisfies all these conditions…" needs reference.

I added a reference.

>24. Line 188: "variable residues during three decades" Which residues are being talked about and the time span? If possible, elaborate a bit more.

I added an explanation for the residues and why those residues seem to have changed.

>25. Line 190: "infectibility" or infectivity?

I wish to thank the reviewer. It was infectibility.

>26. Line 191: “influenza A did.” To be replaced with “influenza A virus did.”

I replaced the wording accordingly.

>27. Line 198: “The ORF lengths for the influenza virus are within a certain range...” What is this range? Add a reference also. This comparison of ORFs must be further expanded for better understanding of the readers.
I have added a reference and explanations.

28. Line 203: “Spike protein… and may cause antibody-dependent enhancement”. Why and how? Must be elaborated here with proper references.

I wish to thank the reviewer for the comment. Here, I cited a wrong reference. I corrected the reference and added a new one related to the phenomenon “antibody dependent enhancement.”

29. The comparison of coronavirus and influenza viruses still requires to be addressed in a more extensive manner in relation to the present findings. For instance, lines 198-203: can an example of influenza virus protein be taken here for comparison?

I wish to thank the reviewer for this comment. Instead of taking a protein as an example, I explained how all proteins changed. In addition, I added information on the length of the ORFs to make the explanations tangible.

30. Lines 224- 228: How can this issue be addressed. Elaborate a bit more and if possible include this in a separate section for conclusion.

I added an explanation for the methodology and explained the real classification of coronaviruses.

Reviewer #2: 1. Title of manuscript is not very apt for the content of manuscript. I would suggest to select more apt title.

I added a term “seasonal” The current title should be suitable for the summary and the concluding paragraph newly placed at the end of the discussion section.

2. Line 33- “Rather, those repeatedly found among humans showed annual changes”. Cite a reference for this sentence.

I have cited a reference here.

3. Line 44- “ a 2010-2015 study in China reported that 2.3% and 30% of patients were positive for coronavirus and
influenza virus, respectively [6]; a similar ratio was found in another large study 46 [6]. Please provide correct referencing for larger study mentioned in the sentence.

I wish to thank the reviewer for this comment. I apologise for the incorrect citation. I corrected it.

4. Full forms of abbreviations used are missing.

I have corrected this accordingly.

5. Materials and methods need extensive revision to indicate type of sample used, which all sequences were compared
and analyzed. Software and tools used to compare and conclude the findings.

All the samples were examined using the same method, as has been written. I added an explanation at the beginning of the paragraph.

6. Line 104-110 sentences need to be reframed and structured for clarity of readers. It

is not clear as a sentence talks
about influenza and suddenly next sentence seems to be talking about coronavirus.

Throughout the Results section, what was discussed was coronavirus. Examples of influenza were used for comparisons with coronavirus. To clarify this, I inserted the term “coronavirus” in every sentence that contains the names of the subclasses in the Results section.

7. Line 114 “Similarly to other RNA viruses [5], many indels were observed, especially in some smaller ORFs” replace similarly with similar.

I replaced the wording accordingly.

8. Many long sentences have been used. For the clarity of readers reframe sentences.

I checked the length of all sentences.
Some were compound sentences. Although they are not harder to understand, I changed those into single sentences (including sentences separated by semicolons).
Some had a non-restrictive relative clause. Those are difficult to separate and should not cause reading difficulty.
The average number of words per sentence was 15.37. This is within the range (12-17) that is asked for scientific writing https://www.aje.com/arc/editing-tip-sentence-length/.
I have re-ordered English editing; I hope this improves the readability.

9. Line 128 “Each of the human-outbreak strains had similar ones in bats or camels, with minor differences 129 (Fig. 1 and S1 Table)”. Please correct it grammatically

I altered the wording.

10. The word contrarily has been used many a times. Use other forms of the word.
The manuscript used the word “contrarily” three times. I have used “in contrast” four times, so maybe this should be avoided. I replaced two instances with “on the other hand” and “to the contrary.”

11. Line 158- These characteristics corroborate the assessment that “coronaviruses can apparently breach cell type, tissue, and host species barriers with relative ease. Relative to what?

The authors of this article did not specify it. However, they compared coronaviruses with influenza or ebola viruses. So, I added “other viruses.”

12. Authors must clearly mention why they are comparing influenza and Coronavirus. How that may help in curbing present pandemic.

I added some information about influenza and quoted a phrase of Sun Tzu.

13. Figures should be discussed extensively in the result and discussion section.

I deleted a part of the Introduction section, which briefly introduced the manuscript; this deleted explanations about the Figures. Some text in the Materials and Methods just show cues to understanding, so they do not use for discussions. I believe that this helps with readability.

14. Results need extensive discussion as at many places information is missing or is not very clear.

I have added some Supporting Figures. I hope that this will improve clarity.

15. Thorough revision of english language and sentence reformation is required.

I requested a professional re-editing of the manuscript. I hope that this will improve the English.

	16. Reference number 15 and 17 need formatting<br><br>I deleted some letters "_" from both of them.
**Additional Information:**	
**Question**	**Response**
**Financial Disclosure**<br><br>Enter a financial disclosure statement that describes the sources of funding for the work included in this submission. Review the submission guidelines for detailed requirements. View published research articles from *PLOS ONE* for specific examples.<br><br>This statement is required for submission and **will appear in the published article** if the submission is accepted. Please make sure it is accurate.<br><br>**Unfunded studies**<br>Enter: *The author(s) received no specific funding for this work.*<br><br>**Funded studies**<br>Enter a statement with the following details:<br>• Initials of the authors who received each award<br>• Grant numbers awarded to each author<br>• The full name of each funder<br>• URL of each funder website<br>• Did the sponsors or funders play any role in the study design, data collection and analysis, decision to publish, or preparation of the manuscript?<br>• **NO** - Include this sentence at the end of your statement: *The funders had no role in study design, data collection and analysis, decision to publish, or preparation of the manuscript.*<br>• **YES** - Specify the role(s) played.<br><br>* typeset	The author(s) received no specific funding for this work.
**Competing Interests**<br><br>Use the instructions below to enter a competing interest statement for this submission. On behalf of all authors, disclose any competing interests that could be perceived to bias this work—acknowledging all financial support	The authors have declared that no competing interests exist.

and any other relevant financial or non-financial competing interests.<br><br>This statement **will appear in the published article** if the submission is accepted. Please make sure it is accurate. View published research articles from *PLOS ONE* for specific examples.<br><br>**NO authors have competing interests**<br><br>Enter: *The authors have declared that no competing interests exist.*<br><br>**Authors with competing interests**<br><br>Enter competing interest details beginning with this statement:<br><br>*I have read the journal's policy and the authors of this manuscript have the following competing interests: [insert competing interests here]*<br><br>* typeset	
**Ethics Statement**<br><br>Enter an ethics statement for this submission. This statement is required if the study involved:<br><br>• Human participants<br>• Human specimens or tissue<br>• Vertebrate animals or cephalopods<br>• Vertebrate embryos or tissues<br>• Field research<br><br>Write "N/A" if the submission does not require an ethics statement.<br><br>General guidance is provided below. Consult the submission guidelines for detailed instructions. **Make sure that all information entered here is included in the Methods section of the manuscript.**	N/A

**Format for specific study types**<br><br>**Human Subject Research (involving human participants and/or tissue)**<br>• Give the name of the institutional review board or ethics committee that approved the study<br>• Include the approval number and/or a statement indicating approval of this research<br>• Indicate the form of consent obtained (written/oral) or the reason that consent was not obtained (e.g. the data were analyzed anonymously)<br><br>**Animal Research (involving vertebrate animals, embryos or tissues)**<br>• Provide the name of the Institutional Animal Care and Use Committee (IACUC) or other relevant ethics board that reviewed the study protocol, and indicate whether they approved this research or granted a formal waiver of ethical approval<br>• Include an approval number if one was obtained<br>• If the study involved *non-human primates*, add *additional details* about animal welfare and steps taken to ameliorate suffering<br>• If anesthesia, euthanasia, or any kind of animal sacrifice is part of the study, include briefly which substances and/or methods were applied<br><br>**Field Research**<br><br>Include the following details if this study involves the collection of plant, animal, or other materials from a natural setting:<br>• Field permit number<br>• Name of the institution or relevant body that granted permission	
**Data Availability**<br><br>Authors are required to make all data underlying the findings described fully available, without restriction, and from the time of publication. PLOS allows rare exceptions to address legal and ethical concerns. See the PLOS Data Policy and FAQ for detailed information.	No - some restrictions will apply

A Data Availability Statement describing where the data can be found is required at submission. Your answers to this question constitute the Data Availability Statement and **will be published in the article**, if accepted.<br><br>**Important:** Stating 'data available on request from the author' is not sufficient. If your data are only available upon request, select 'No' for the first question and explain your exceptional situation in the text box.<br><br>Do the authors confirm that all data underlying the findings described in their manuscript are fully available without restriction?	
**Describe where the data may be found in full sentences. If you are copying our sample text, replace any instances of XXX with the appropriate details.**<br><br>• If the data are **held or will be held in a public repository**, include URLs, accession numbers or DOIs. If this information will only be available after acceptance, indicate this by ticking the box below. For example: *All XXX files are available from the XXX database (accession number(s) XXX, XXX.)*.<br>• If the data are all contained **within the manuscript and/or Supporting Information files**, enter the following: *All relevant data are within the manuscript and its Supporting Information files.*<br>• If neither of these applies but you are able to provide **details of access elsewhere**, with or without limitations, please do so. For example:<br><br>*Data cannot be shared publicly because of [XXX]. Data are available from the XXX Institutional Data Access / Ethics Committee (contact via XXX) for researchers who meet the criteria for access to confidential data.*<br><br>*The data underlying the results presented in the study are available from (include the name of the third party*	[Note to PA: Please confirm DAS w AUs and ask if they received special access privileges for their third party data]<br>Most relevant data are available in the Supporting Information files. However, data from GISAID are available at https://www.gisaid.org/.

and contact information or URL).<br>• This text is appropriate if the data are owned by a third party and authors do not have permission to share the data.<br><br>* typeset	
Additional data availability information:	

Powered by Editorial Manager® and ProduXion Manager® from Aries Systems Corporation

Manuscript Click here to access/download;Manuscript;Manuscript.docx

Principal component analysis of coronaviruses reveals their diversity and seasonal and pandemic potential.

Tomokazu Konishi[1]*

Short title: Coronaviruses, a source of pandemic and seasonal pathogens

[1] Faculty of Bioresource Sciences, Akita Prefectural University, Akita, Japan

*Corresponding author

E-mail: konishi@akita-pu.ac.jp

Abstract

Coronaviruses and influenza viruses have similarities and differences. In order to comprehensively compare them, their genome sequencing data were examined by principal component analysis. Coronaviruses had fewer variations than a subclass of influenza viruses. In addition, differences among coronaviruses that infect a variety of hosts were also small. These characteristics may have facilitated the infection of different hosts. Although many of the coronaviruses were conservative, those repeatedly found among humans showed annual changes. If SARS-CoV-2 changes its genome like the Influenza H type, it will repeatedly spread every few years. In addition, the coronavirus family has many other candidates for new pandemics.

Introduction

Coronavirus disease 2019 (COVID-19) is rapidly spreading worldwide [1, 3]. To investigate its spreading mechanism, genomes of coronaviruses were analysed by principal component analysis (PCA) [4] and compared to influenza viruses [5]. Both coronaviruses and influenza viruses cause annual epidemics and pandemics; however, they differ in host specificity and the rate of mutations. Comparing the diversity and changes in their genomes will help to understand the characteristics of the present pandemic. Such characteristics would be useful for confronting the virus.

Influenza and coronaviruses have RNA genomes. Both genomes directly replicate using their RNA-dependent RNA polymerases, which may lead to many errors [6]. This characteristic has introduced variations among the viruses, including the number and size of their open reading frames (ORFs) [7]. These viruses have different lengths of genome segments. While the

coronavirus genome is nearly 30 kb long [2, 8], the influenza genome is divided into eight segments, and the total length is nearly 14 kb [9]. Hence, compensation for the different lengths is required to facilitate comparison; to fulfil this, the values of the PCA were scaled [10].

Some classes of coronaviruses, such as human coronavirus (HCoV), cause upper respiratory tract infections in humans. The symptoms are similar to those of the common cold, although they may also cause severe pneumonia [11]. They have lower infectivity than human influenza viruses. For example, a 2010-2015 study in China reported that 2.3% and 30% of patients were positive for coronavirus and influenza virus, respectively; a similar ratio was found in another large study [12]. Some of the strains of coronaviruses may have much higher infectivity and cause outbreaks, such as severe acute respiratory syndrome (SARS)-CoV [13, 14], Middle East Respiratory Syndrome (MERS)-CoV [13, 15, 16], and SARS-CoV-2 (SCoV2) [1, 3, 17, 18]. The former two cause severe symptoms, while the latter varies from asymptomatic to critical. However, although the fatality rate of COVID-19 is still being estimated, it could become five times higher than that of seasonal influenza [19] causing many complications [20]. Additionally, COVID-19 is thought to be more contagious among certain populations and age groups than influenza [20].

The corona and influenza viruses have similarities and differences in infectivity, spreadability, and symptomatology. These differences are based on their genomes, which are important for estimating how SCoV2 will act in humans.

Materials and Methods

Data and classification

All nucleotide sequences were analysed using the same method [4]. The method gives principal components (PCs) in a form that is scaled for the length of sequences or the number of virus samples. Sequencing data were obtained from the DNA Data Bank of Japan (DDBJ) database [21]. Aligned data, obtained with DECIPHER [22] (presented in the Supplementary material S1-S3 data), were further processed to observe the relationships among virus samples by using the direct PCA method [4], which can handle data under the least assumptions. The conceptual diagram of the PCA is shown in the next section (all calculations were performed in R) [23] and updated versions of the scripts can be found in GitHub (https://github.com/TomokazuKonishi/direct-PCA-for-sequences). To avoid the imbalance effect among samples, decomposition was performed by removing clusters of similar samples, for example, clusters of sequences from SARS, MERS, or SCoV2. Instead, only one sample was included from each cluster. Aligned sequences of the virus samples are available in the Supporting material (S1-S3 data).

To prepare a comprehensive data set for SCoV2, 2796 full-length sequences were obtained from the Global Initiative on Sharing All Influenza Data (GISAID) database [24] and added to those used for Fig. 1. Some records were preliminary and contained many uncertain bases (designated by “N”), which may be counted as indels. To cancel such artefacts, the corresponding regions were replaced with the average data, which cancels the corresponding bases from the results of the PCA. The list of subjected sequences is available in S3 data.

Diagram of the PCA

sequencing data matrix (nucleotide/amino acids)

↓ Numerical conversion

boolean matrix

↓ Finding the data centre and subtraction

centred matrix

↓ Singular value decomposition

scaled PC for base and samples

To enable calculations, the sequence data is translated into numerals to create a matrix of samples and bases [4]. The matrix is then rotated by the PCA, and the centre of the rotation is the mean sequence. The direction of rotation is determined by the directions of the differences: if distinguishing variations exist and they spread the samples into two groups, the variations will define a direction. If another set of variations separates the samples into a third and fourth groups, then the variations define another direction. Then, an axis is set toward a direction. The rotated data are dispersed among the axes. The axis has a sensitivity to specific nucleotide base positions, and each sample shows a specific PC for the axis. Hence, the virus samples are separated on the axis in accordance with the type of base the sample has. As all the axes are orthogonal, a smaller number of axes extract the differences efficiently. Singular value decomposition determines the direction of the axis. As the translation and rotation are reversible, the original sequences can be restored from the PCs. No unverifiable assumptions were used. These characteristics—preservation of information and falsifiability—are quite different from hierarchical clustering methods. Here, I presented the first two axes, which covered 10 % to 30

% of the differences in the data. The PCs were scaled to enable comparisons among different nucleotide lengths or sample numbers [10].

Estimation of the magnitude of sample variations

The variation magnitude among sample sequences was estimated by the mean distances, scaled by the length of the sequence m, of virus types. This was a sort of standard deviation, $\bar{d} = \sqrt{\sum(x_i - \bar{x})^2/2nm}$, where x_i is the Boolean of each sample sequence, $\bar{x}$ is the mean sample, and n is the number of samples. Coefficient 2 was used to correct the double counts of the differences. The unit of length is the same as that of the PCA, which will extract the length toward particular directions.

Estimation of mutation levels in the genome

The levels of PCs 1-5 for bases were estimated by the root sum square at each base position (Fig. 2 and S3). If there are alterations in several samples, and if they occur coincidently, they may contribute to a higher level of PC. To see the tendencies at the positions, two moving averages with a width of 200 amino acid residues were shown for substitutions (grey) and indels (blue).

Results

The coronaviruses separated into distinct classes (Fig. 1), which could be further divided into subclasses (S1 Fig.). For example, SARS-CoV and SCoV2 belong to different subclasses of Sarbecovirus (S1 Fig. and S1 Table). The origin of the graph (0, 0) coincides with the mean data.

The accumulation of mutations form a variety of viruses that have different directions and distances from the original (ancestral) virus. If the mutations and samplings are random, the original virus will be near the data mean.

Fig. 1. Classes of coronaviruses separated into PC1 and 2. Blue: human samples; subclasses of HCoV are indicated. a: Alphacoronavirus; d: Delta coronavirus; D: Duvinacovirus; e: Embecovirus; g: Gammacoronavirus; m: Merbecovirus; n: Nobecovirus; s: Sarbecovirus; T: TGEV. A major estimated class, Betacoronavirus, is not shown here, since the class has to cover distinctive classes (Embecovirus, Merbecovirus, Norbecovirus, and Sarbecovirus). It should be noted that many of the classifications in the original records were different from those obtained in this study.

The variation magnitude, estimated by the mean distance $\bar{d}$, was 0.11. This is much smaller than those of single subclasses of influenza A virus (S2 Fig.), such as H1 or H9. The value has been scaled so it has a kind of generality; actually, the value of $\bar{d}$ was not significantly altered by artificial reductions of sample numbers or sequence length (not shown).

Among the classes of the coronaviruses, Gammacoronavirus and Deltacoronavirus, which are close to the origin of the graph, were mainly found in bird samples (Fig. 1 and S1 Table). These could be the origin of coronaviruses, as like the influenza viruses are thought to have originated from those of waterfowls. The bat coronavirus Norbecovirus, which seems to be the origin of the mammalian viruses, represented the mean of the studied samples. Indeed, many other classes were found in bat samples. The most distant classes from the mean, TGEV (Transmissible gastroenteritis virus) and Embecovirus, were found in larger animals, but not in bats.

Strains of HCoV belonged to Embecovirus, Alphacoronavirus, and Dubinacovirus (Fig. 1, blue). On the other hand, the strains of recent major epidemics originated from two classes without common cold viruses: Merbecovirus and Sarbecovirus.

Similar to other RNA viruses [6], many indels were observed, especially in some smaller ORFs (Fig. 2 and S3). The indels ranged from small regions without frameshifts to large ones that alter multiple ORFs, for example Embecovirus is unique because it possesses an ORF of hemagglutinin. The class is further distinguished by having another ORF, NS2a, which can also not be present (Figs 1 and 2). Even within a small group of HCoVs, OC43, there was an indel corresponding to 14 amino acids in the spike protein. The classification was not significantly affected by focusing on the indels or on the rest of the sequences (S4 Fig.). Therefore, indels were not given extra weight in this study; they were treated as a base or a residue. Note that some small ORFs, such as the envelope and nucleocapsid, are conservative and lack indels.

Fig. 2. Frequency of mutations in each position of the genome. The level is estimated by the root sum square of PC1-PC5 (grey circles). The moving averages are represented in a 1:5 scale. The names of the ORFs are indicated. The indel at NS2a reflects that there are subclasses with the ORF and without the ORF.

The values of PCs were not significantly affected by the hosts (Fig. 1). For example, differences between bird and swine coronaviruses in Deltacoronaviruses were small (S1 Table, PC18). This is in contrast to influenza viruses, which were separated among different hosts. For example, in influenza H1N1, the waterfowl class is near the centre, with three swine groups around it, and two human groups further apart (S2 Fig.) [5]. For coronaviruses, those that are more distant from Norbecovirus seem to infect larger animals, but this rule is not absolute (S1 Table).

Each of the human epidemic coronaviruses had similar viruses in bats or camels, although there were minor differences (Fig. 1 and S1 Table). In the SARS-CoV spike protein, no amino acid residue was unique to humans. This is partially because our knowledge about the viruses has increased after the efforts to screen for likely viruses in wild animals [2, 25-27]. Only 35 out of 2412 residues were different between SARS-CoV and similar bat viruses, and many of these were not conserved among the bat samples (S2 Table). The situation was the same with SCoV2, which presented 34 unique amino acid residues (S2 Table); naturally, this uniqueness will be reduced after further research.

The year occurrence of influenza A H1N1 and HCoVs is very different, since only one H1N1 variety spreads worldwide yearly (S2G Fig.) [5], while several OC43 variants appear even within a single country (Fig. 3A, S5, and S3 Table). H1 variants will never return in the subsequent seasons, whereas OC43 varieties appear repeatedly for a decade. However, by concentrating solely on one variety, the annual alterations became obvious (Fig. 3B). As an example, a variety with a high PC1 value was selected, which was found in 1985-2000 in the USA and in 2002 in France (Fig. 3A). The mutation speed was much slower than that of influenza H1N1 (S2 G Fig.); as the PC values are scaled, the magnitudes can be compared directly.

Fig. 3. A. PC of the reported HCoV OC43 spike protein in each year. The letters represent the countries/regions: C- China, E- EU, M- Malaysia, U- USA. The upper-most series of variants were selected for a focused view (next panel). **B**, annual changes in the selected variants. Similar one-directional changes found in PC1 were always found in influenza H1N1 human cases [3]. **C**.

Classes of SCoV2 separated in PC1 and 2. Data from 2836 samples are shown. Examples of countries are indicated. The full set of records is presented in Table S4.

A comprehensive set of SCoV2 samples was separated into three directions, forming some classes (Fig. 3C, S6A). These could result from the spread of a mutated virus in a previously virus-free population. Various classes were found in some countries, suggesting multiple influx routes. The class closest to the data mean was that of China. Samples reported from countries far from China tend to show larger magnitudes of PCs, and vice versa (S4 Table).
Shift-type alterations are frequently found in the influenza virus [5], which could be caused by exchanging RNA segments among any two influenza viruses. This type of alteration was also observed in coronaviruses, even though the genome was not segmented. By focusing on the spike protein, coronaviruses separated (Fig. 4A) in a similar way to the classification obtained by the whole genome (Fig. 1). However, in the classification obtained by the 1ab polyprotein of coronavirus, the positions of Deltacoronavirus and Embecobirus were exchanged (Fig. 4B). Additionally, the position of the nucleocapsid protein in SARS-CoV moved from OC43, losing Embecovirus unity (Fig. 4C). These drastic changes are difficult to explain without shifts.

Fig. 4 A. Classification obtained from the amino acid sequences of the spike protein. The relationships between the classes were similar to those estimated from the entire nucleotide sequences (Fig. 1). **B**. Classes found in polyprotein 1ab. The positions of the Deltacoronavirus and the Sarbecovirus were replaced. **C**. Classes found in the nucleocapsid protein. SARS-CoV moved out of the Sarbecovirus class.

Discussion

The coronavirus and influenza classes were fairly different in two aspects: although the groups were clearly separated, the differences did not match those of the hosts in coronavirus, and the divergence magnitude among coronaviruses was much lower than that of a subclass of the influenza A virus. These characteristics corroborate the assessment that "coronaviruses can apparently breach cell type, tissue, and host species barriers with relative ease" compared to other viruses [28]. Spike protein-mediated infection by coronavirus would be more tolerant to differences in hosts than the influenza virus haemagglutinin protein, which may have eased the selective pressure to separate the groups of coronaviruses according to the various hosts. Shifts, which are not possible by replacing virus segments, may have occurred in coronaviruses. Since these viruses do not copy the genome into double-stranded DNA, RNA splicing in the nucleus [29] or RNA interference would be used instead of the ordinal homologous recombination by the RecA family [30]. This may also be the cause of the frequent indels. Viruses change their genome according to various selective pressures [6], such as to: a) Maintain functions: any changes can cause malfunctions; hence, they have to conserve the genome, which is required for any state of the virus. b) Escape herd immunity: Influenza A viruses, which are highly infectious, escape herd immunity by continuously changing. All ORFs change at the same speed [5], as any part of the virus that could be represented by the major histocompatibility complex will become a target of the immune system. In contrast, HCoV is not very infectious; hence, some people remain unimmunised, easing this pressure. Additionally, the resulting smaller number of replications per year would slow the rate of changes. This might be the case for MERS-CoV among camels [15, 31, 32]. c) Exploit new hosts: this may require a change in the docking system. Adaptation to the genetic system of a new host may alter codon usage and

several amino acids [33]. SARS-CoV and SCoV2 might be under this type of pressure during infection. d) Increase asymptotic patients: patients with mild or no symptoms are required for survival of the virus. In humans, once all infected individuals are identified, the virus is contained, especially if the symptoms are critical.

For influenza viruses the conditions required to cause a pandemic are obvious. First, it has to be highly infective, such as type H1N1. Second, it should be free from herd immunity. For example, Pdm09 belongs to one of the two subclasses that did not cause outbreaks among humans [5]. The SARS- and MERS-CoV fulfilled these conditions, but they failed to escape the selective pressure mentioned above in (d). SCoV2 satisfies all these conditions, thus it is spreading worldwide as Pdm09 did [3].

For the past three decades the dominant R type H1 of influenza A changed annually [5] by changing its most variable residues, the outermost surface amino acids of the protein structure. In contrast, the inner core region of the protein was conserved; this suggests that the selective pressure is in escaping immunity. The new Pdm09 is also changing annually. Coronaviruses have shown few annual changes (Fig. 3B and S6A), which might be due to their limited infectibility (HCoV) or the lack of infected people (MERS-CoV). SCoV2 will face the selective pressure of herd immunity (b) as influenza A did. If it escapes this selective pressure, it will remain among humans and spread every few years. Actually, the change in SCoV2 has already begun; they have formed several classes within a short emergence time (Fig. 3C, 6B). The magnitudes of the PCs may indicate the migration pathways of the classes. They might mutate within China, transfer to other countries and mutate further (S6 Fig.). These changes may help acclimatisation to humans (c); however, they may also relate to herd immunity (b) and/or lower lethality (d).

Fortunately, the lifespan of the classes of coronaviruses should be shorter than those of influenza viruses. The ORF lengths for influenza viruses are within a range of 2.3k-0.9Kb [9] and all ORFs change annually at the same rate [5]. In contrast, some of the coronavirus ORFs are quite short. For example, the envelope protein [35] is only 260 bases long and located in the conserved region of the genome [34] (Fig. 2 and S3). This protein might be too short to form a variable structure, making it a good target for herd immunity. These conservative ORFs might be suitable vaccine targets. In contrast, the spike protein tended to change (S3 Fig.) and may cause antibody-dependent enhancement (ADE), since it covers the surface of the virus. In ADE, the antibody that binds to the virus may help it enter the target cell through Fc or complement receptors [36] (in addition to the Ace2/Spike protein receptors [37]). There is evidence that this occurs with the SARS-CoV spike protein [38] and there is concern about SCoV2 [39]. Influenza proteins showed annual changes at the same rate, showing that any of the viral components would be under the same selective pressure [5]. If this is also true in coronaviruses, recognising proteins other than the spike protein will help with understanding the immune mechanisms.

Many bat coronaviruses seemed to be able to infect humans. The bat and human viruses are similar (Fig. 1), and there are more variations in bats, of which we have observed only a part. Due to replication errors and RNA editing, a bat may possess several variants of coronavirus [6]. Although host-virus specificities are shown in laboratory experiments [28], as humans and other animals have individual variations, the barrier would be more fragile in reality. Once an infection occurs, the virus will adapt through mutations [27]. Long-term accumulation of mutations in intermediate hosts, such as pigs for influenza viruses, is not essential. These viruses would have a limited infectious character and cause mild symptoms to bats or other hosts; however, they could show an excessive adaptation to humans as SARS- and MERS-CoV. As humans do not

have herd immunity to many classes of coronaviruses (other than HCoV), these might produce a new pandemic.

If intermediates are required, the main contribution could be the amplification of the inoculum size and contact frequency. Bats and human habitats are different, and a bat may not produce enough viral particles to infect different hosts. The size of the donor animal is important, for example, healthcare workers with secondary MERS infections tend to have milder symptoms and a better prognosis [32]. This could be caused by differences in inoculum sizes; as camels produce nasal secretions full of viruses [31, 41], the inoculum size would be larger than that from humans. To prevent the infection of intermediate animals, live animals of different species should not be kept in the same place. Additionally, identifying the first patients is essential to prevent a human outbreak. Thus, people who have frequent contact with wildlife should not live in a cosmopolitan city.

The conventional classification system separates coronavirus into four major groups, from alpha to delta [8]; these classes have been found by a clustering method. It is true that the methodologies always find a tree structure for the relationships; however, they are not suitable for estimating the classification of many data. For example, they cannot accurately depict the relationship between distant samples (they only show the directly connected ones). Additionally, they do not suggest the root of the tree. Furthermore, they depend on many assumptions that cannot be verified. In the case of coronaviruses, the four-group structure was far from the results we found by PCA; for example, the categories of Alpha- and Beta-coronavirus actually contained large variations. Additionally, many of the credits for the classification of original sequencing records were misjudged (this was also true for influenza viruses). Estimating the classification using phylogenetic methods has poor reproducibility, and misjudgements would

have been caused by such difficulty. Using an objective method is preferable to determine the attributions [4]. One of the advantages of PCA is that it can apply a classification to sets of other data without affecting the established one. This will facilitate finding attributions of new samples.

As a source of pandemic viruses, coronaviruses have many candidates that are new to humans, and can become seasonal viruses, with annual mutations. Here, the nucleotide sequences of coronaviruses were evaluated using direct PCA [4]. The variations in coronavirus were much smaller than in influenza and differences among hosts were also small (Fig. 1). There were several classes; some of them included human viruses, but others did not. SARS, MERS, and COVID-19 belonged to a class that lacked HCoVs. Some of the ORFs in coronavirus are rather conservative, and hence would be ideal targets for vaccines (Fig. 2). HCoVs are more conservative than influenza (Fig. 3A and S2G), but they also show annual changes (Fig. 3B). SARS-CoV-2 has changed during spreading to the world; it will continue mutating if it stays as a pandemic virus.

Acknowledgments

The author would like to thank Editage (www.editage.com) for English language editing.

References

1. Yuen K-S, Ye ZW, Fung S-Y, Chan C-P, Jin D-Y. SARS-CoV-2 and COVID-19: The most important research questions. Cell & Bioscience. 2020;10(1):40. doi: 10.1186/s13578-020-00404-4.
2. Rota PA, Oberste MS, Monroe SS, Nix WA, Campagnoli R, Icenogle JP, et al. Characterization of a Novel Coronavirus Associated with Severe Acute Respiratory Syndrome. Science. 2003;300(5624):1394-9. doi: 10.1126/science.1085952.
3. WHO. Coronavirus disease (COVID-19) Pandemic 2020 [cited 2020 23 May]. Available from: https://www.who.int/emergencies/diseases/novel-coronavirus-2019.
4. Konishi T, Matsukuma S, Fuji H, Nakamura D, Satou N, Okano K. Principal Component Analysis applied directly to Sequence Matrix. Scientific Reports. 2019;9(1):19297. doi: 10.1038/s41598-019-55253-0.

5. Konishi T. Re-evaluation of the evolution of influenza H1 viruses using direct PCA. Scientific Reports. 2019;9(1):19287. doi: 10.1038/s41598-019-55254-z.
6. Agol VI, Gmyl AP. Emergency Services of Viral RNAs: Repair and Remodeling. Microbiology and molecular biology reviews : MMBR. 2018;82(2):e00067-17. doi: 10.1128/MMBR.00067-17. PubMed PMID: 29540453.
7. Lauring AS, Frydman J, Andino R. The role of mutational robustness in RNA virus evolution. Nature reviews Microbiology. 2013;11(5):327-36. Epub 2013/03/26. doi: 10.1038/nrmicro3003. PubMed PMID: 23524517; PubMed Central PMCID: PMCPMC3981611.
8. Woo PC, Huang Y, Lau SK, Yuen KY. Coronavirus genomics and bioinformatics analysis. Viruses. 2010;2(8):1804-20. Epub 2010/08/01. doi: 10.3390/v2081803. PubMed PMID: 21994708; PubMed Central PMCID: PMCPMC3185738.
9. McCauley JW, Mahy BW. Structure and function of the influenza virus genome. Biochem J. 1983;211(2):281-94. doi: 10.1042/bj2110281. PubMed PMID: 6191756.
10. Konishi T. Principal component analysis for designed experiments. BMC Bioinformatics. 2015;16 Suppl 18:S7. doi: 10.1186/1471-2105-16-S18-S7. PubMed PMID: 26678818; PubMed Central PMCID: PMCPMC4682404.
11. Talbot HKB, Crowe Jr. JE, Edwards KM, Griffin MR, Zhu Y, Weinberg GA, et al. Coronavirus infection and hospitalizations for acute respiratory illness in young children. Journal of Medical Virology. 2009;81(5):853-6. doi: 10.1002/jmv.21443.
12. Zhang S-f, Tuo J-l, Huang X-b, Zhu X, Zhang D-m, Zhou K, Yuan L, Luo H-j, Zheng B-j, Yuen K-y, Li M-f, Cao K-y, Xu L. Epidemiology characteristics of human coronaviruses in patients with respiratory infection symptoms and phylogenetic analysis of HCoV-OC43 during 2010-2015 in Guangzhou. PLoS ONE 2018;13(1): e0191789. doi: https://doi.org/10.1371/journal.pone.0191789.
13. Song Z, Xu Y, Bao L, Zhang L, Yu P, Qu Y, et al. From SARS to MERS, Thrusting Coronaviruses into the Spotlight. Viruses. 2019;11(1):59. doi: 10.3390/v11010059. PubMed PMID: 30646565.
14. WHO. Severe Acute Respiratory Syndrome (SARS) 2020 [cited 2020 23 May]. Available from: https://www.who.int/csr/sars/en/.
15. Dawson P, Malik MR, Parvez F, Morse SS. What Have We Learned About Middle East Respiratory Syndrome Coronavirus Emergence in Humans? A Systematic Literature Review. Vector-Borne and Zoonotic Diseases. 2019;19(3):174-92. doi: 10.1089/vbz.2017.2191. PubMed PMID: 30676269.
16. WHO. Middle East respiratory syndrome coronavirus (MERS-CoV) 2020 [cited 2020 23 May]. Available from: https://www.who.int/emergencies/mers-cov/en/.
17. Wu F, Zhao S, Yu B, Chen Y-M, Wang W, Song Z-G, et al. A new coronavirus associated with human respiratory disease in China. Nature. 2020;579(7798):265-9. doi: 10.1038/s41586-020-2008-3.
18. Zhou P, Yang X-L, Wang X-G, Hu B, Zhang L, Zhang W, et al. A pneumonia outbreak associated with a new coronavirus of probable bat origin. Nature. 2020;579(7798):270-3. doi: 10.1038/s41586-020-2012-7.
19. Faust JS, del Rio C. Assessment of Deaths From COVID-19 and From Seasonal Influenza. JAMA Internal Medicine. 2020;180(8):1045-6. doi: 10.1001/jamainternmed.2020.2306.
20. CDC. Similarities and Differences between Flu and COVID-19 2020. Available from: https://www.cdc.gov/flu/symptoms/flu-vs-covid19.htm.
21. Ogasawara O, Kodama Y, Mashima J, Kosuge T, Fujisawa T. DDBJ Database updates and computational infrastructure enhancement. Nucleic acids research. 2020;48(D1):D45-d50. Epub 2019/11/15. doi: 10.1093/nar/gkz982. PubMed PMID: 31724722; PubMed Central PMCID: PMCPMC7145692.
22. Wright ES. DECIPHER: harnessing local sequence context to improve protein multiple sequence alignment. BMC Bioinformatics. 2015;16:322. Epub 2015/10/09. doi: 10.1186/s12859-015-0749-z. PubMed PMID: 26445311; PubMed Central PMCID: PMCPMC4595117.
23. R Core Team. R: A language and environment for statistical computing. Vienna, Austria: R Foundation for Statistical Computing; 2017.
24. Elbe S, Buckland-Merrett G. Data, disease and diplomacy: GISAID's innovative contribution to global health. Global challenges (Hoboken, NJ). 2017;1(1):33-46. doi: 10.1002/gch2.1018. PubMed PMID: 31565258.
25. The Chinese SARS Molecular Epidemiology Consortium. Molecular Evolution of the SARS Coronavirus During the Course of the SARS Epidemic in China. Science. 2004;303(5664):1666. doi: 10.1126/science.1092002.
26. Guan Y, Zheng BJ, He YQ, Liu XL, Zhuang ZX, Cheung CL, et al. Isolation and Characterization of Viruses Related to the SARS Coronavirus from Animals in Southern China. Science. 2003;302(5643):276-8. doi: 10.1126/science.1087139.

27. Lu H, Zhao Y, Zhang J, Wang Y, Li W, Zhu X, et al. Date of origin of the SARS coronavirus strains. BMC Infectious Diseases. 2004;4(1):3. doi: 10.1186/1471-2334-4-3.
28. Graham RL, Baric RS. Recombination, Reservoirs, and the Modular Spike: Mechanisms of Coronavirus Cross-Species Transmission. Journal of Virology. 2010;84(7):3134-46. doi: 10.1128/jvi.01394-09.
29. Zhou C, Liu S, Song W, Luo S, Meng G, Yang C, et al. Characterization of viral RNA splicing using whole-transcriptome datasets from host species. Scientific reports. 2018;8(1):3273-. doi: 10.1038/s41598-018-21190-7. PubMed PMID: 29459752.
30. Haber JE. DNA Repair: The Search for Homology. BioEssays : news and reviews in molecular, cellular and developmental biology. 2018;40(5):e1700229-e. Epub 2018/03/30. doi: 10.1002/bies.201700229. PubMed PMID: 29603285.
31. Adney DR, Bielefeldt-Ohmann, H., Hartwig, A. E., & Bowen, R. A. . Infection, Replication, and Transmission of Middle East Respiratory Syndrome Coronavirus in Alpacas. Emerging Infectious Diseases. 2016;22(6):1031-7.
32. Dawson P, Malik MR, Parvez F, Morse SS. What Have We Learned About Middle East Respiratory Syndrome Coronavirus Emergence in Humans? A Systematic Literature Review. Vector-Borne and Zoonotic Diseases. 2019;19(3):174-92. doi: 10.1089/vbz.2017.2191. PubMed PMID: 30676269.
33. Bahir I, Fromer M, Prat Y, Linial M. Viral adaptation to host: a proteome-based analysis of codon usage and amino acid preferences. Mol Syst Biol. 2009;5:311-. Epub 2009/10/13. doi: 10.1038/msb.2009.71. PubMed PMID: 19888206.
34. Surya W, Li Y, Torres J. Structural model of the SARS coronavirus E channel in LMPG micelles. Biochimica et Biophysica Acta (BBA) - Biomembranes. 2018;1860(6):1309-17. doi: https://doi.org/10.1016/j.bbamem.2018.02.017.
35. Sarkar M, Saha S. Structural insight into the role of novel SARS-CoV-2 E protein: A potential target for vaccine development and other therapeutic strategies. PLoS ONE 2020;15(8):e0237300. doi: https://doi.org/10.1371/journal.pone.0237300.
36. Takada A, Kawaoka Y. Antibody-dependent enhancement of viral infection: molecular mechanisms and in vivo implications. Reviews in medical virology. 2003;13(6):387-98. Epub 2003/11/20. doi: 10.1002/rmv.405. PubMed PMID: 14625886.
37. Li W, Moore MJ, Vasilieva N, Sui J, Wong SK, Berne MA, et al. Angiotensin-converting enzyme 2 is a functional receptor for the SARS coronavirus. Nature. 2003;426(6965):450-4. doi: 10.1038/nature02145.
38. Yip MS, Cheung CY, Li PH, Bruzzone R, Peiris JSM, Jaume M. Investigation of Antibody-Dependent Enhancement (ADE) of SARS coronavirus infection and its role in pathogenesis of SARS. BMC Proc. 2011;5(Suppl 1):P80-P. PubMed PMID: PMC3019510.
39. Arvin AM, Fink K, Schmid MA, Cathcart A, Spreafico R, Havenar-Daughton C, et al. A perspective on potential antibody-dependent enhancement of SARS-CoV-2. Nature. 2020. doi: 10.1038/s41586-020-2538-8.
40. Lau SKP, Woo PCY, Li KSM, Huang Y, Tsoi H-W, Wong BHL, et al. Severe acute respiratory syndrome coronavirus-like virus in Chinese horseshoe bats. Proceedings of the National Academy of Sciences of the United States of America. 2005;102(39):14040-5. doi: 10.1073/pnas.0506735102.
41. Adney DR, van Doremalen N, Brown VR, Bushmaker T, Scott D, de Wit E, et al. Replication and shedding of MERS-CoV in upper respiratory tract of inoculated dromedary camels. Emerging infectious diseases. 2014;20(12):1999-2005. doi: 10.3201/eid2012.141280. PubMed PMID: 25418529.

Supporting information

S1 Fig. Classes of coronaviruses at the presented axes of PCs. Blue: human samples. Labels are the same as in Fig. 1c. Classes found in the Sarbecovirus. SARS-CoV and SCoV2 belong to different groups.

S2 Fig. Subclasses of Influenza A virus. a. H1 hemagglutinin, **b**. H4 PB1, **c**. H5 hemagglutinin, **d**. H7 PB2, **e**. H9 hemagglutinin, **f**. H9 PB1. Values of the mean distance were indicated. The subclass may coincide with the hosts (**d**) but in many cases, one host species formed a distinct class.

S3 Fig. Levels of PC1-PC5 at each position in the nucleotide sequences. a. Sarbecovirus, **b**. HCoV OC43, **c**. MERS-CoV. **d**. SCoV2. Names of the ORFs are indicated in Fig. 2.

S4 Fig. Separation of classes in PC1 and 2. Estimated using indels (**a**) and substitutions (**b**).

S5 Fig. Subclasses of OC43 were separated by the indicated PCs found in the whole genome. Conservative characteristics of the virus and repeated appearance were obvious.

S6 Fig. a Annual changes in the MERS-CoV genome. b Comprehensive data for SARS-CoV-2. Samples found in some European countries showed higher magnitudes of PCs, indicating accumulation of mutations (also see Table S4).

S1 Table. PC for samples of whole genome sequences of coronaviruses. Figure 1 was made from a part of this table.

S2 Table. Differences in spike proteins a. SARS-CoV and bat SARS-like viruses b. SCoV2 and other coronaviruses. Aligned protein sequences were compared. The IDs for the samples are listed.

S3 Table. PC for samples of HCoV OC43 coronaviruses.

S4 Table. PCs of SARS-CoV-2.

S1 data. Aligned sequences for the coronaviruses.

S2 data. Aligned sequences for the OC43.

S3 data. Aligned sequences for SARS-CoV-2.

Fig4

Click here to access/download;Figure;Fig4.tif

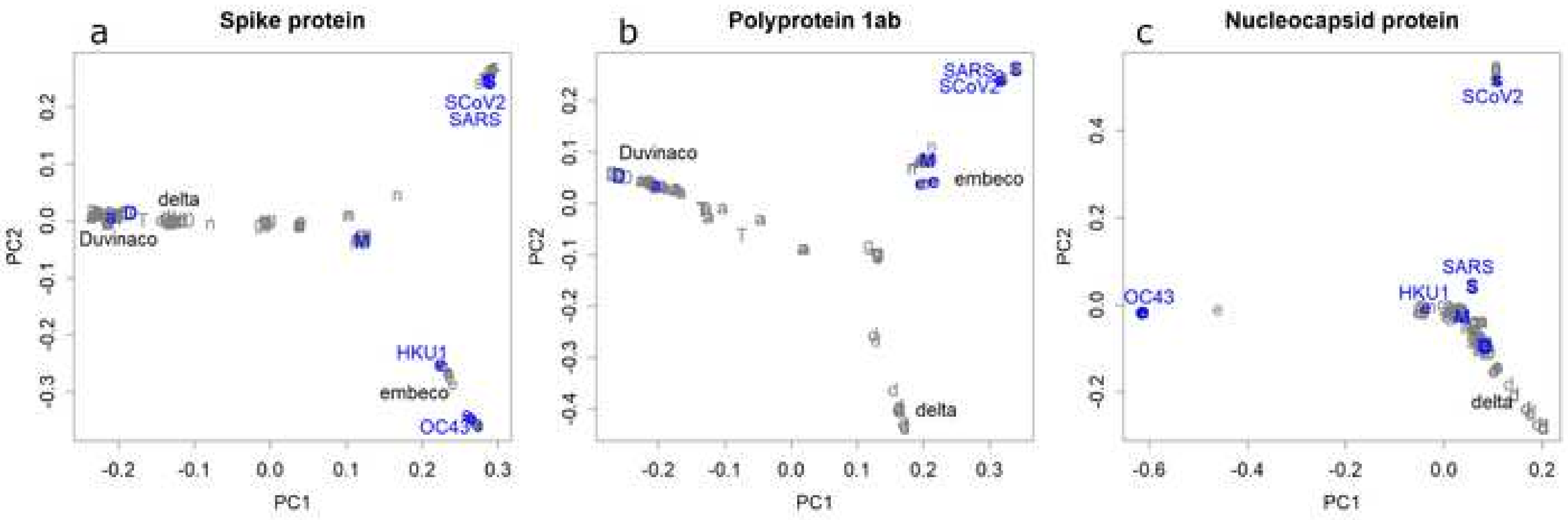

Fig3

Click here to access/download;Figure;Fig3.tif

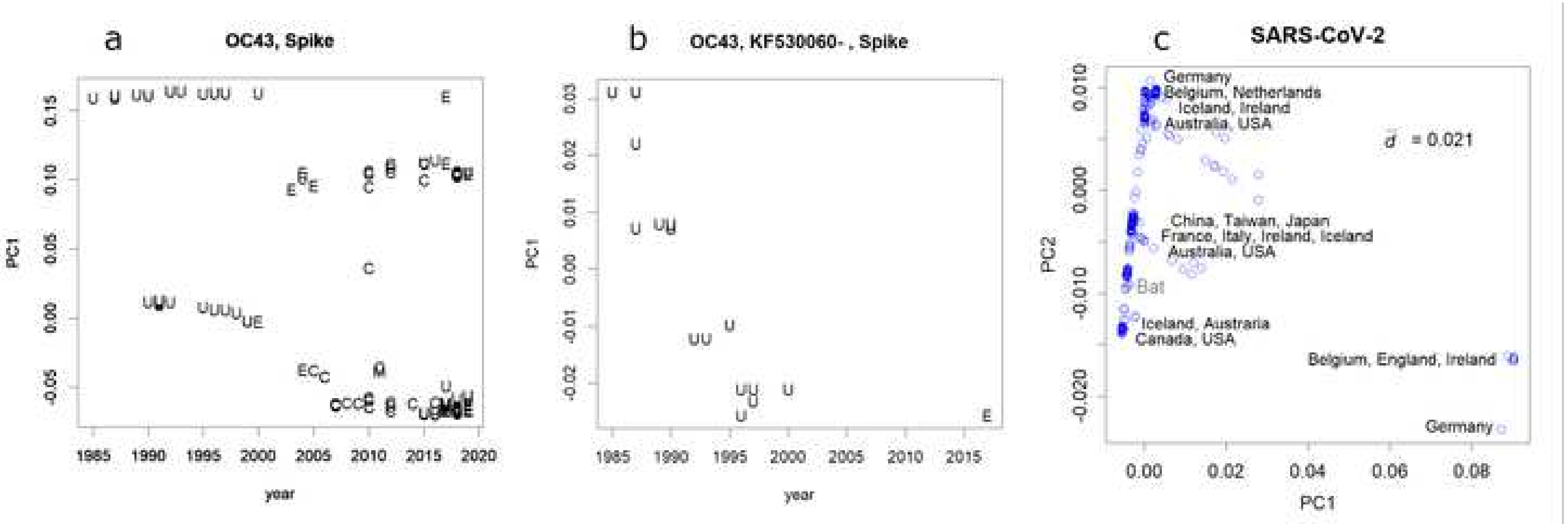

Fig2

Click here to access/download;Figure;Fig1.tif

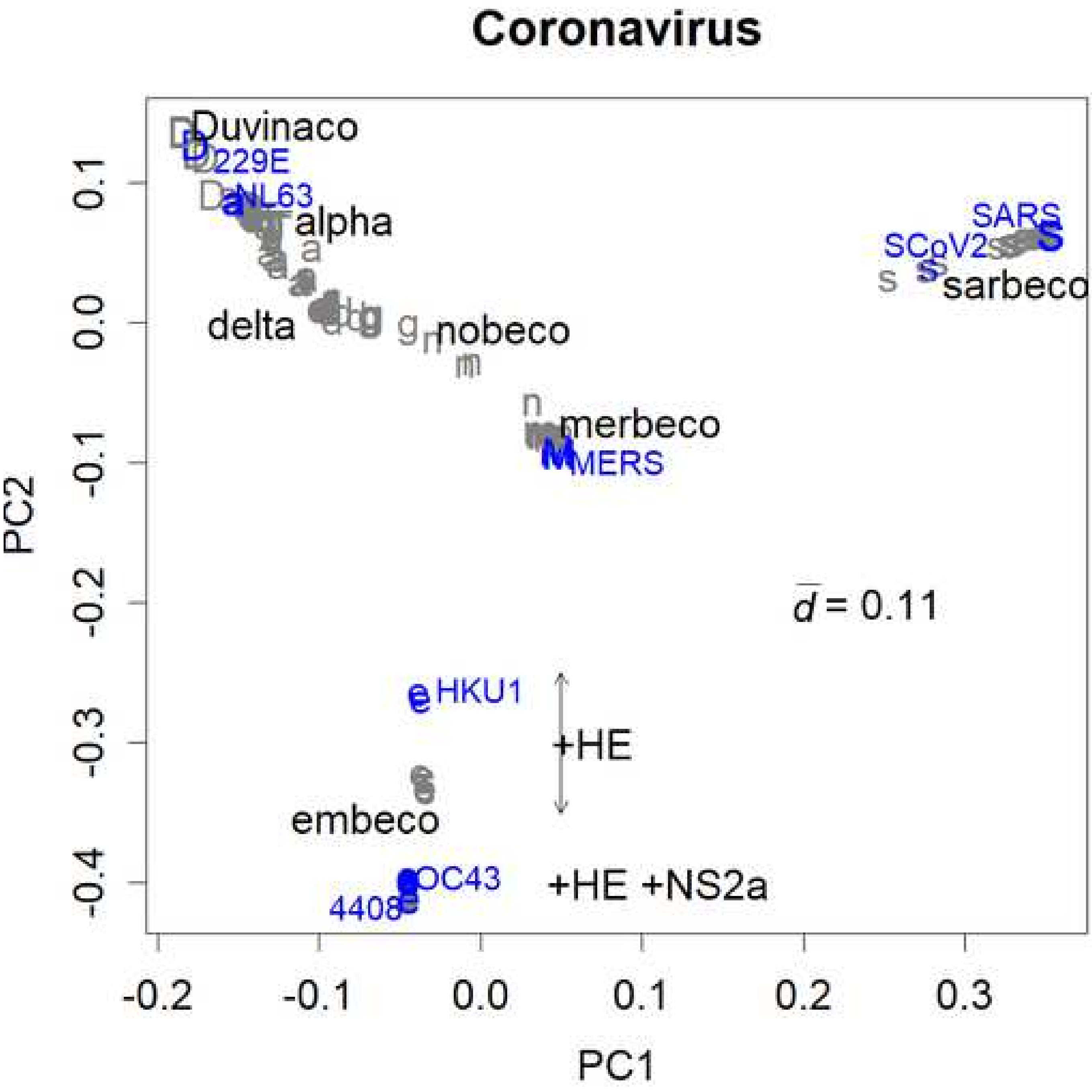

 Click here to access/download;Figure;Fig2.tif

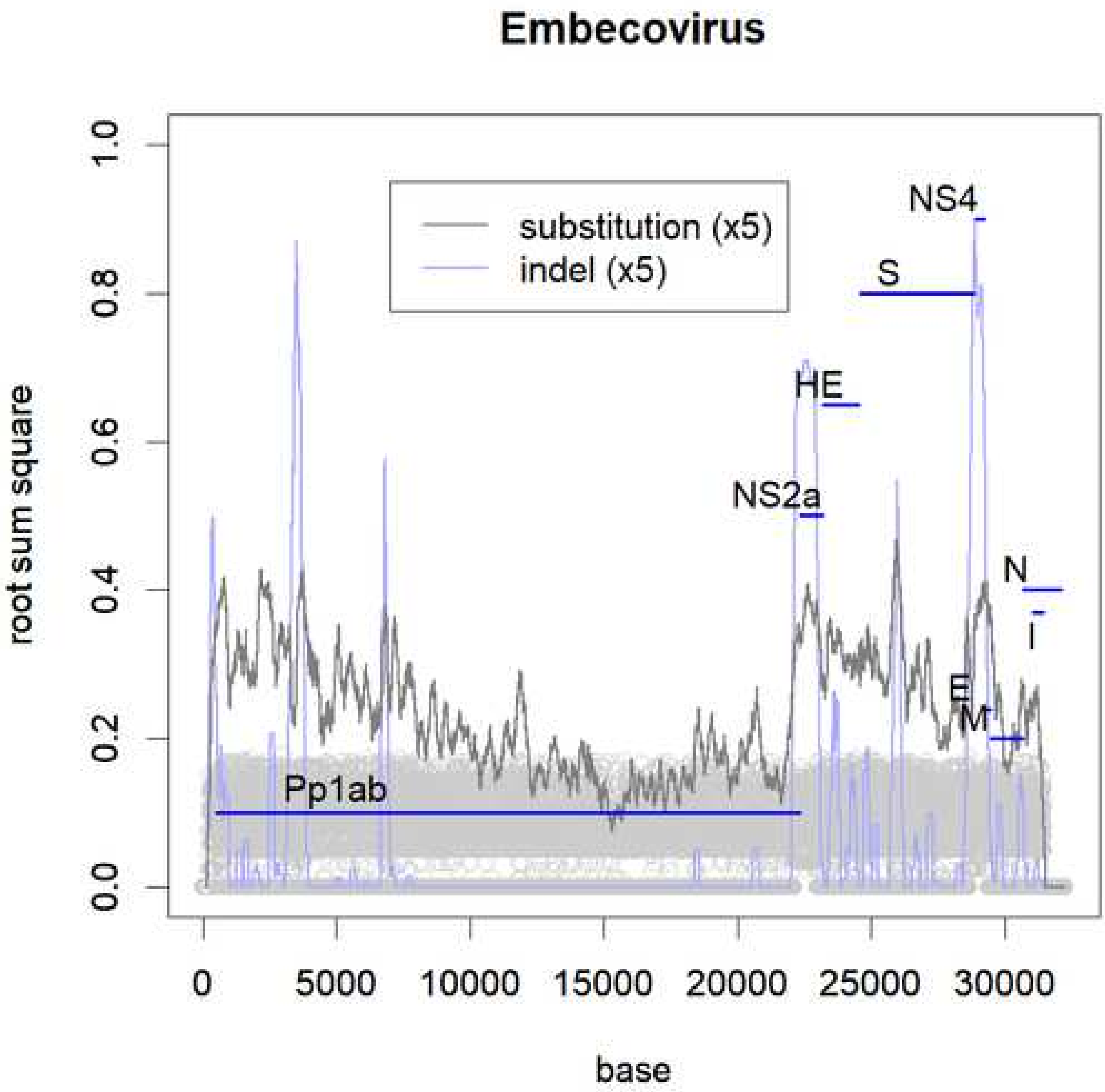

Click here to access/download
Supporting Information - Compressed/ZIP File Archive
supporting.zip

Principal component analysis of coronaviruses reveals their diversity and seasonal and pandemic potential.

Tomokazu Konishi[1]*

Short title: Coronaviruses, a source of pandemic and seasonal pathogens

~~Affiliations:~~

[1] Faculty of Bioresource Sciences, Akita Prefectural University, Akita, Japan

*Corresponding author

E-mail: konishi@akita-pu.ac.jp

Formatted: English (United Kingdom)

Formatted: English (United Kingdom)

Formatted: English (United Kingdom), Superscript

Formatted: English (United States)

Formatted: Head

Formatted: English (United Kingdom)

Formatted: English (United Kingdom)

Formatted: English (United Kingdom)

Formatted: English (United States)

Formatted: English (United Kingdom)

Formatted: Line spacing: Double

Abstract

The cCoronavirusescoronavirus and the influenza virusesvirus have similarities and differences. In order to comprehensively compare them, their genome sequencing data were examined by principal component analysis. Variations in cCoronaviruses had lessfewer variationscoronavirus were smaller than those in a subclass of the influenza virusesvirus. In addition, differences among coronaviruses that infectin a variety of hosts were also small. These characteristics may have facilitated the infection of different hosts. Although many of the coronaviruses were more conservative, those repeatedly found among humans showed annual changes. If SARS-CoV-2 changes its genome like the Influenza H type, it will repeatedly spread every few years. In addition, the coronavirus family has many other candidates for newsubsequent pandemics.

Introduction

Coronavirus disease 2019 (COVIDCOVID (coronavirus disease)-19) [1] COVID-19 [1, 2] is rapidly spreading worldwide [1, 3]. To investigate its spreading mechanism, genomes of the coronaviruses were analysedanalyzed by principal component analysis (PCA) [4] and compared to those of the influenza viruses [5]. Both coronaviruses and influenza virusesof these viruses cause annual epidemics and pandemics; however, they differ in host specificity and the speedrate of mutations. Comparing the diversityies and changeschanging inof their genomes will help to understand the characteristics of the present pandemic. Such characteristics., which Such characteristics would be useful for confronting the virus.

Influenza and coronaviruses have RNA genomes. Both genomes and. Both are directly replicated using their RNA-dependent RNA polymerases, which may causelead to many errors [6]. This

Formatted: English (United Kingdom)

Formatted: English (United Kingdom)

Formatted: English (United Kingdom)

Formatted: English (United Kingdom)

Formatted: English (United Kingdom)

Formatted: English (United Kingdom)

Formatted: English (United Kingdom)

Formatted: Font: 12 pt, English (United Kingdom)

Formatted: English (United Kingdom)

characteristic has introduced variations among the viruses, including the number and size of their open reading frames (ORFs) [7]. These~~Those~~ viruses have~~are~~ different ~~in the~~ lengths~~length~~ of ~~the~~ genome segments~~;~~. ~~w~~While the coronavirus genome is nearly 30 kb long~~RNA~~ [2, 8], the influenza genome is divided into~~in~~ eight segments, and the total length is nearly 14 kb [9]. Hence, compensation for the different lengths~~length~~ is required to facilitate ~~the~~ comparison~~s~~; to fulfil this, the values of the PCA were scaled [10].

Some classes of coronaviruses, such as human coronavirus (HCoV), cause upper respiratory tract infections in humans. The symptoms are ~~mostly~~ similar to those of the common cold, although they may also cause severe pneumonia [11]. They have lower infectivity than ~~the~~ human influenza viruses. For example, a 2010-2015 study in China reported that 2.3% and 30% of patients were positive for coronavirus and influenza virus, respectively; a similar ratio was found in another large study [12]. Some of the strains of coronaviruses may have ~~a~~ much higher infectivity and cause outbreaks~~: e.g.~~, such~~SARS~~ as ~~(~~severe~~Severe~~ acute respiratory syndrome (SARS)-CoV [13, 14], ~~MERS (~~Middle East Respiratory Syndrome (MERS)~~-~~-CoV [13, 15, 16], and SARS-CoV-2 (SCoV2) [1, 3, 17, 18]. The former two cause severe symptoms, while the latter varies from asymptomatic to critical. However, although the fatality rate of COVID-19 is still being~~under the process of~~ estimated~~ing~~, it could~~would~~ become five times ~~more~~higher than that of seasonal influenza [19] ~~by~~ causing many complications [20].~~;~~ Additionally~~also~~, COVID-19 is thought to be more contagious among certain populations and age groups than influenza [20].

~~is rapidly spreading worldwide. To investigate its spreading mechanism, genomes of the coronavirus were compared to those of the influenza virus [5] by principal component analysis (PCA) [4]. These two RNA viruses differed in host specificity and speed of mutations. Here, I~~

present the characteristics of both and the changes in coronavirus. The coronaviruses presented smaller variations and did not differ much from host to host. These characteristics may have eased the novel transfection of coronaviruses to humans; many of the bat strains seem to have the ability to infect humans, and the intermediate animals may not be necessarily required, against the present expectations. Although many of the coronaviruses were more conservative, their character may reflect a less selective pressure owing to their limited infectivity. Rather, those repeatedly found among humans showed annual changes. If SARS-CoV-2, which is highly infectious, changes its genome like the influenza H type, it will repeatedly spread every few years. In addition, the coronavirus family may have other candidates for subsequent outbreaks. While the coronavirus genome is a positive-sense single-stranded 30 kb RNA, the influenza genome is divided in eight segments. Both directly replicate using their RNA-dependent RNA polymerases, which may cause many errors [6]. This characteristic has introduced variations among coronaviruses, including the number and size of their open reading frames (ORFs). Some classes of coronaviruses, such as HCoV, cause upper respiratory tract infections in humans. The symptoms are mostly similar to those of the common cold, but they may also cause severe pneumonia [11]. They have lower infectivity than the human influenza viruses. For example, a 2010-2015 study in China reported that 2.3% and 30% of patients were positive for coronavirus and influenza virus, respectively [11]; a similar ratio was found in another large study [11]. Some viruses may have a much higher infectivity and cause outbreaks: e.g., SARS-CoV [13, 14], MERS-CoV [13, 15, 16], and SARS-CoV-2 (SCoV2) [1, 3, 17, 18]. The former two cause severe symptoms, while the latter varies from asymptomatic to critical.

The corona and influenza viruses have similarities and differences in infectivity, spread-ability, and symptomatologys.~~symptoms,~~ These differences are based on their genomes, which are important for estimating how SCoV2 will act in humans.

Formatted: English (United Kingdom)

Formatted: Font: English (United Kingdom)

Materials and Methods

Data and classification

All ~~the~~ nucleotide sequences were analysed~~analyzed~~ using~~by~~ the~~a~~ same method [4]. The method gives principal components (PCs) in a form that is scaled for the length of sequences or the number of virus samples. Sequencing data were obtained from ~~DDBJ~~ the ~~(~~DNA Data~~data~~ Bank~~bank~~ of Japan (DDBJ) database [21]. Aligned data, obtained with DECIPHER [22] (presented in the Supplementary material S1-S3 data), were further processed to observe the relationships among virus samples by using the direct PCA method [4], which can handle data under the least assumptions. The conceptual diagram of the PCA is shown in the next section~~as follows~~ (all calculations were performed in R) [23]~~.~~ and ~~U~~updated versions of the scripts ~~are presented~~can be found in GitHub (https://github.com/TomokazuKonishi/direct-PCA-for-sequences). To ~~escape~~avoid the imbalance effect among samples, ~~the~~ decomposition was performed by removing clusters of similar samples, for example,~~e.g.~~ ~~those~~clusters of sequences from ~~caused by~~ SARS, MERS, or SCoV2. Instead, only one sample was included from each cluster. Aligned sequences of the ~~subjected~~ virus samples are available in the Supporting material (S1-S3 data).

~~Sequencing data were obtained from DDBJ database. Aligned data, obtained with DECIPHER~~[22] ~~(presented in the Supplementary material), were further processed to observe~~

~~the relationships among samples by using the direct PCA method [4], which can handle data with limited assumptions. The conceptual diagram of the PCA is as follows (all calculations were performed in R)~~ [23]~~. Updated versions of the scripts are presented in GitHub (https://github.com/TomokazuKonishi/direct-PCA-for-sequences). To escape the imbalance effect among samples, the decomposition was performed by removing clusters of similar samples, e.g. those caused by SARS, MERS, or SCoV2. Instead, only one sample was included from each cluster.~~

To prepare a comprehensive data set for SCoV2, 2~~,~~796 ~~of~~ full-length ~~data~~sequences ~~-~~were obtained from the Global Initiative on Sharing All Influenza Data (GISAID) ~~-~~database [24] and added to those used for Fig. 1. Some ~~of those~~ records were ~~rather~~ preliminary and contained many~~several~~ uncertain bases (designated by “N”)~~,”~~, which may be counted as indels. To cancel such artefacts~~artifacts~~, the corresponding regions were replaced with the average data, ~~;~~ which~~this~~ cancels the corresponding bases from the results of the ~~in the~~ PCA. The list of subjected sequences is available in ~~the Supporting material~~ S3 data.

Formatted: English (United Kingdom)
Formatted: English (United Kingdom)
Formatted: English (United Kingdom)
Formatted: English (United Kingdom)
Formatted: English (United Kingdom)
Formatted: English (United Kingdom)
Formatted: English (United Kingdom)
Formatted: English (United Kingdom)
Formatted: English (United Kingdom)
Formatted: English (United Kingdom)
Formatted: English (United Kingdom)

Diagram of the PCA

Formatted: English (United Kingdom)

sequencing data matrix (nucleotide/amino~~Nucleotides / Amino~~ acids~~Acids~~)

↓ Numerical conversion

boolean matrix

↓ ~~f~~Finding~~finding~~ the data ~~centerecentre~~centre and subtraction

centred matrix

↓ Singular value decomposition

scaled PC for base and samples

Formatted: English (United Kingdom)
Formatted: English (United Kingdom)
Formatted: English (United Kingdom)
Formatted: English (United Kingdom)

To enable calculations, the sequence data is translated into numerals to create~~make~~ a matrix of samples and bases [4]. The matrix is then rotated by the PCA,~~;~~ and the ~~center~~centre of the rotation is the mean sequence. The direction of rotation is determined by the directions of the differences: if distinguishing variations exist and they spread the samples into two groups, the variations will define a direction. If another set of variations separates~~separate~~ the samples into ~~the~~a third and fourth groups, then the variations define another direction. Then, an axis is set toward a direction. The rotated data are~~will be~~ dispersed among the axes~~axis~~. The axis has a sensitivity to specific nucleotide base positions, and each sample shows a specific PC for the axis. Hence, the virus samples are separated on the axis in accordance with the~~which~~ type~~kind~~ of base the sample has. As all the axes are orthogonal, a smaller number of axes extract the differences efficiently. Singular value decomposition determines~~finds~~ the direction of the axis. As the translation and ~~the~~ rotation are reversible, the original sequences can be restored from the ~~found~~ PCs. No unverifiable assumptions were~~are~~ used. These characteristics—~~ – ~~preservation of information and falsifiability—~~ – ~~are quite different from hierarchical clustering methods. Here, I ~~mainly~~ presented the first two axes, which covered ~~3~~10 % to ~~1~~30 % of ~~data~~the differences in the data. The ~~found~~ PCs were scaled to enable comparisons among different nucleotide lengths~~length~~ or sample numbers [10].

Estimation of the magnitude of ~~the~~ sample variations

The variation magnitude~~scale~~ among sample sequences was estimated by the mean distances, scaled by the length of the sequence m, of virus types. This was a sort~~type~~ of standard deviation, $\bar{d} = \sqrt{\sum(x_i - \bar{x})^2/2nm}$ ~~$\sqrt{\sum(x - \bar{x})^2/2nm}$~~, where x_i is ~~x, $\bar{x}$, and n are~~ the Boolean

Formatted: SM Text

Formatted: English (United Kingdom)

Formatted: English (United Kingdom)

Formatted: English (United Kingdom)

Formatted: English (United Kingdom)

Formatted: English (United Kingdom)

Formatted: English (United Kingdom)

Formatted: English (United Kingdom)

Formatted: English (United Kingdom)

Formatted: English (United Kingdom)

of each sample sequence, $\bar{x}$ is the mean sample, and n is the number of samples~~, respectively~~. Coefficient 2 was used to correct the double counts of the differences. The unit of length is the same as that of the PCA, which will extract the length toward particular directions.

Formatted: Font: Italic, English (United Kingdom)
Formatted: English (United Kingdom)
Formatted: Font: Italic, English (United Kingdom)
Formatted: English (United Kingdom)
Formatted: English (United Kingdom)
Formatted: English (United Kingdom)

Estimation of mutation levels in the genome

The levels of PCs 1-5 for bases were estimated by the root sum square at each base position (Fig. 2 and S3). If there are alterations ~~existed~~ in several samples, and if they occur coincidently, they may contribute to a higher level of PC. To see the tendencies at the positions, two moving averages with a width of 200 amino acid residues were shown for substitutions (grey) and indels (blue).

Formatted: English (United Kingdom)

Results

The coronaviruses separated into distinct classes (Fig. 1), which could be further divided into subclasses (S1 Fig.). ~~The coronaviruses separate intoconsist of distinct classes (Fig. 1). In the lower PC axes, the oAnother set ofother classes were separated in the lower PC axis (S1 Fig.), and these were further divided into subclasses.~~ For example, SARS-CoV and SCoV2 belong to different subclasses of -Sarbecovirus (S1 Fig. and S1 Table). The origin of the graph~~,~~ (0, 0) coincides with the mean data. The accumulation of mutations ~~will~~ form a variety of viruses~~,~~ that~~which~~ have different directions and distances from the~~an~~ original (ancestral) virus. If the mutations and samplings are random, the original virus will~~ould~~~~would~~ be near ~~to~~ the data mean.

Formatted: English (United Kingdom)
Formatted: English (United Kingdom)
Formatted: Font: English (United Kingdom)
Formatted: Font: English (United Kingdom)
Formatted: English (United Kingdom)
Formatted: Font: English (United Kingdom)
Formatted: Font: English (United Kingdom)
Formatted: Font: English (United Kingdom)
Formatted: Font: English (United Kingdom)
Formatted: Font: 10 pt, English (United States)

Fig. 1. Classes of coronaviruses separated into PC1 and 2. Blue: human samples; subclasses of HCoV are indicated. a: Alphacoronavirus; d: Delta coronavirus; D: Duvinacovirus; e: Embecovirus; g: Gammacoronavirus; m: Merbecovirus; n: Nobecovirus; s: Sarbecovirus; T: TGEV. A major estimated class, Betacoronavirus, is not shown here, since the class has to cover distinctive classes (Embecovirus, Merbecovirus, Norbecovirus, and Sarbecovirus). It should be noted that many of the classifications ~~credits~~ in the original records were different from those obtained in this study~~presentation~~.

Formatted: English (United Kingdom)

The variation magnitude, estimated by the mean distance $\bar{d}$, was 0.11. This is much smaller than those of single subclasses of influenza A virus (S2 Fig.), such as H1 or H9. The value has been scaled so it has a kind of generality; actually~~Incidentally~~, the value of $\bar{d}$ was not significantly altered by artificial reductions of sample numbers or sequence length (not shown).

Formatted: English (United Kingdom)

Formatted: English (United Kingdom)

Formatted: English (United Kingdom)

Formatted: English (United Kingdom)

Formatted: English (United Kingdom)

Formatted: English (United Kingdom)

Among the classes of the coronaviruses, Gammacoronavirus and Deltacoronavirus, which are close to the origin of the graph, were mainly found ~~mainly~~ in bird samples (Fig. 1 and S1 Table). These could be the origin of coronaviruses, as like ~~like~~ the influenza viruses are thought to have originated from those of waterfowls. The ~~mean of the studied samples was located in the~~ bat coronavirus~~virus~~ Norbecovirus, which seems to be the origin of the mammalian viruses ~~in mammals~~, represented the mean of the studied samples.~~,~~ Indeed, many other classes ~~apart from this class~~ were found in bat samples. The most distant classes from the mean, TGEV (Transmissible gastroenteritis virus) and Embecovirus, were found in larger animals, but not in bats.

Formatted: English (United Kingdom)

Formatted: English (United Kingdom)

Formatted: English (United Kingdom)

Formatted: English (United Kingdom)

Formatted: English (United Kingdom)

Formatted: English (United Kingdom)

Formatted: English (United Kingdom)

Formatted: English (United Kingdom)

Strains of ~~Human coronaviruses (~~HCoV belonged~~) belong~~ to Embecovirus, Alphacoronavirus, and Dubinacovirus (Fig. 1, blue). On the other hand, the strains of recent major epidemics ~~were~~

Formatted: English (United Kingdom)

Formatted: English (United Kingdom)

originated from two~~Two~~ classes without ~~such ~~common cold viruses:~~,~~ Merbecovirus and Sarbecovirus~~, originated in recent outbreaks~~.

Similar to other RNA viruses [6], many indels were observed, especially in some smaller ORFs (Fig. ~~Similarly to other RNA viruses ~~[6]~~, many indels were observed, especially in some smaller ORFs (Fig. 2 and S3). These range from small regions without frameshifts to large ones that alter plural ORFs, e.g.~~2 and S3). The indels ranged from small regions without frameshifts to large ones that alter ~~plural~~multiple ORFs, for example~~e.g.~~ Embecovirus is unique because it possesses an ORF of hemagglutinin. The class is further distinguished by having another ORF, NS2a, which can also~~or~~ not be present (Figs 1 and 2). Even within a small group of HCoVs~~HCoV~~, OC43, there was an indel ~~of~~corresponding to 14 amino acids~~aa length existed~~ in the spike protein. The classification was not significantly affected by ~~either ~~focusing on the indels or on the rest of the sequences (S4 Fig.). Therefore, indels were not given extra weight in this study; they were treated as a base or a residue. Note that some ~~other ~~small ORFs, such as the envelope and nucleocapsid, are conservative and lack indels.

Fig. 2. Frequency of mutations in each position of the genome. The level is estimated by the root sum square of PC1-PC5 (grey circles). The moving averages are represented in a 1:5 scale. The names of the ORFs are indicated. The indel at NS2a reflects that there are subclasses with the ORF and without the ORF.

The values of PCs~~PC~~ were not significantly affected by the hosts (Fig. 1).~~)~~ ~~e.g. ~~For example, differences between bird and swine coronaviruses~~viruses~~ in Deltacoronaviruses were small (S1 Table, PC18). This is in contrast~~contrarily~~ to influenza viruses, which were separated among different hosts. For example, in influenza H1N1~~ of influenza~~, the waterfowl~~; the~~ class ~~for~~

Formatted: English (United Kingdom)
Formatted: English (United Kingdom)
Formatted: English (United Kingdom)
Formatted: English (United Kingdom)
Formatted: English (United Kingdom)
Formatted: English (United Kingdom)
Formatted: English (United Kingdom)
Formatted: English (United Kingdom)
Formatted: English (United Kingdom)
Formatted: English (United Kingdom)
Formatted: English (United Kingdom)
Formatted: English (United Kingdom)
Formatted: English (United Kingdom)
Formatted: English (United States)
Formatted: English (United Kingdom)
Formatted: Normal
Formatted: English (United Kingdom)
Formatted: English (United Kingdom)
Formatted: English (United Kingdom)
Formatted: English (United Kingdom)
Formatted: English (United Kingdom)
Formatted: English (United Kingdom)

~~waterfowl~~ is ~~located~~ near the centre, with three swine groups ~~located~~ around it, and two human groups further~~were positioned in the most~~ apart (S2 Fig.) [5]. ~~In~~For coronaviruses, those that are more distant[5]~~. In coronaviruses, those~~ apart from ~~the~~ Norbecovirus seem to infect larger animals, but this rule is not absolute (S1 Table).

Each of the human epidemic ~~human~~ coronaviruses~~coronavirus outbreak strains~~ had similar ~~ones~~viruses in bats or camels, although there were~~with~~ minor differences (Fig. 1 and S1 Table). In the SARS-CoV spike protein ~~of SARS-CoV~~, no amino acid residue was unique to humans. This is partially because our knowledge about the viruses has increased after the efforts to screen for likely viruses in wild animals [2, 25-27]. Only 35 out of 2412 residues were different ~~from~~between SARS-CoV and similar bat viruses, and many of these were not conserved among the bat samples (S2 Table). The situation was the same ~~in~~with SCoV2, which presented 34 unique amino acid residues (S2 Table); naturally~~however~~, this uniqueness will be reduced~~could disappear~~ after further research.

The year occurrence of ~~I~~influenza~~Influenza~~ A H1N1 and HCoVs~~HCoV~~ ~~yearly occurrence~~ is very different, since only one H1N1 variety spreads worldwide yearly (S2G Fig.) [5]~~. On the other hand~~, while[5]~~. Contrarily,~~ several OC43 variants appear even within a single country (Fig. 3A~~3a~~, S5, and S3 Table). H1 variants will never return in the subsequent seasons, whereas OC43 varieties appear~~appeared~~ repeatedly for a decade. However, by concentrating solely on one variety, the annual alterations became obvious (Fig. 3B). As an example, ~~here the~~a variety with a high PC1 value was selected~~.~~, ~~It~~which was found in 1985-2000 in the USA and in 2002 in France (Fig. 3A). The mutation speed ~~of alteration~~ ~~is~~was much slower than that of influenza~~Influenza~~ H1N1 (S2 G Fig.); as the PC values are scaled, the magnitudes can be compared directly.~~3b).~~

Formatted: English (United Kingdom)
Formatted: English (United Kingdom)
Formatted: English (United Kingdom)
Formatted: English (United Kingdom)
Formatted: English (United Kingdom)
Formatted: Font: English (United Kingdom)
Formatted: Font: English (United Kingdom)
Formatted: English (United Kingdom)
Formatted: English (United Kingdom)
Formatted: English (United Kingdom)
Formatted: English (United Kingdom)
Formatted: English (United Kingdom)
Formatted: English (United Kingdom)
Formatted: English (United Kingdom)
Formatted: English (United Kingdom)
Formatted: English (United Kingdom)
Formatted: English (United Kingdom)
Formatted: English (United Kingdom)
Formatted: English (United Kingdom)
Formatted: English (United States)
Formatted: English (United Kingdom)
Formatted: Normal

Fig. 3. A. PC of the reported HCoV OC43 spike protein in each year. The letters represent the countries/regions: C- China, E- EU, M- Malaysia, U- USA. The upper-most series of variants were ~~further~~ selected for a~~to~~ focused view~~on them in the~~ (next panel). **B**, annual changes in the selected variants. Similar one-directional changes found in PC1 were always found in influenza H1N1 human cases [3]. **C**. Classes of SCoV2 separated in PC1 and 2. Data from 2~~,~~836 samples are shown. Examples of countries are indicated. The full set of records is presented in Table S4.

A comprehensive set of SCoV2 samples was separated into three directions, forming some classes (Fig. 3C, S6A). These~~3c, S6a). Such classes~~ could result from the spread of a mutated virus in a previously virus-free population~~be made if a few persons with mutated virus migrated to another place and the virus started to infect people~~. ~~Plural~~Various~~. Plural~~ classes were found in some countries, suggesting multiple influx routes. The class closest to the data mean was that of China. Samples reported from ~~the~~ countries far from China tend to show larger magnitudes of PCs, and vice versa (S4 Table).

Shift-type alterations are~~were~~ frequently found in the influenza virus [5]~~,;~~ which~~this~~ could~~would~~ be caused by exchanging RNA segments among any two influenza viruses. This type of alteration was also~~were~~ observed in coronaviruses, even though the genome was~~is~~ not segmented.~~separated. This is contrastive to the cases of influenza, which can replace RNA molecules between viruses.~~ By focusing on the spike protein, coronaviruses ~~were~~ separated (Fig. 4A) in a~~4a)~~ similar~~ly~~ way to the classification obtained by the whole genome (Fig. 1). However, in the classification obtained by the 1ab polyprotein of coronavirus, the positions of Deltacoronavirus and Embecobirus were exchanged (Fig. 4B~~4b~~). Additionally, the position of

Formatted: English (United Kingdom)

Formatted: English (United Kingdom)

Formatted: English (United Kingdom)

Formatted: English (United Kingdom)

Formatted: English (United Kingdom)

Formatted: English (United Kingdom)

Formatted: Font: English (United Kingdom)

Formatted: English (United Kingdom)

Formatted: English (United Kingdom)

Formatted: English (United Kingdom)

Formatted: English (United Kingdom)

Formatted: English (United Kingdom)

Formatted: English (United Kingdom)

the nucleocapsid protein in SARS-CoV moved from OC43, losing the EmbecovirusEmvecovirus unity (Fig. 4C4c). These drastic changes are difficult to explain without shifts.

Fig. 4 A. Classification obtained from the amino acid sequences of the spike protein. The relationships between the classes were similar to those estimated from the entire nucleotide sequences (Fig. 1). **B**. Classes found in polyprotein 1ab. The positions of the Deltacoronavirus and the Sarbecovirus were replaced. **C**. Classes found in the nucleocapsid protein. SARS-CoV moved out of the Sarbecovirus class.

Discussion

The coronavirusCoronavirus and influenza classes were fairly different in the following two aspects: although the groups were clearly separated, the differences did not match those of the hosts in coronavirus,; and the divergence magnitude among coronaviruses was much lower than that of a subclass of the influenza A virus. These characteristics corroborate the assessment that “coronaviruses can apparently breach cell type, tissue, and host species barriers with relative ease” compared to other viruses [28]. The Spike[28]. Coronavirus spike protein-mediatedspike protein mediated infection by coronavirus would be more tolerant to differences in hosts than the influenza virus' haemagglutininhemagglutinin protein, which may have eased the selective pressure to separate the groups of coronaviruses according to the various hosts.

Shifts, which are not possible by replacing virus segments, may have occurred in coronaviruses. Since these virusesit does not copy the genome into double-stranded DNA, RNA splicing in the

Formatted: English (United Kingdom)
Formatted: English (United Kingdom)
Formatted: English (United States)
Formatted: English (United Kingdom)
Formatted: Normal
Formatted: English (United Kingdom)
Formatted: Teaser
Formatted: English (United Kingdom)
Formatted: English (United Kingdom)
Formatted: English (United Kingdom)
Formatted: English (United Kingdom)
Formatted: English (United Kingdom)
Formatted: English (United Kingdom)
Formatted: English (United Kingdom)

nucleus [29] or RNA interference would be used instead of the ordinal homologous recombination by the RecA family [30]. This~~Those~~ may also be the cause of the frequent indels. ~~Shifts, which are not possible by replacing virus segments, may have occurred in coronavirus. Since it does not copy the genome into double-stranded DNA, RNA splicing in nucleus~~ [29] ~~or RNA interference would be used instead of the ordinal homologous recombination by the RecA family~~ [30]~~. Those may also be the cause of frequent indels.~~

Viruses change their genome according to various selective pressures [6], such as to: a) Maintain ~~the~~ functions: any changes can cause malfunctions; hence, [6]~~, such as to: a) Maintain the functions: any changes can cause malfunctions~~, ~~hence~~ they have to conserve the genome~~s~~, which is required for any state of the virus. b) Escape ~~the~~ herd immunity: Influenza A viruses, which are highly infectious, escape herd immunity by continuously changing. All ORFs change at the same speed [5], as any part of the virus that could be represented by the major histocompatibility complex will become a target of the immune system. In contrast~~To the contrary~~ ~~Contrarily~~, HCoV is not very infectious; hence, some people remain unimmunised~~unimmunized~~, easing this pressure. ~~Also~~Additionally, the resulting smaller number of replications per year would slow the ~~speed~~ rate of changes. This might be the case for MERS-CoV among camels ~~also~~ [15, 31, 32]. c) Exploit new hosts: this may require a change in the docking system. Adaptation to the genetic system of a new host may alter codon usage and several amino acids [33].~~,~~ SARS-CoV and SCoV2 might be under this type of pressure during infection. d) Increase asymptotic patients: patients with mild or no symptoms are required for ~~transmission success~~survival of the virus.~~,~~ In humans, once all infected individuals are identified, the virus is contained, especially if the symptoms are critical.

Formatted: English (United Kingdom)

Formatted: English (United Kingdom)

Formatted: English (United Kingdom)

Formatted: English (United Kingdom)

Formatted: English (United Kingdom)

Formatted: English (United Kingdom)

Formatted: English (United Kingdom)

Formatted: English (United Kingdom)

For influenza viruses ~~T~~the~~The~~ conditions required to cause a pandemic are obvious~~ in cases of influenza viruses~~. First, it has to be highly infective~~,~~ such as type H1N1. Second, it should be free from herd immunity. For example, ~~the~~ Pdm09 belongs to one of the two subclasses that did not cause outbreaks among humans [5].[5]~~.~~ The SARS- and MERS-CoV fulfilled~~fulfil~~ these conditions, but they failed to escape the selective pressure mentioned above in (d). SCoV2 satisfies all these conditions, thus it is spreading worldwide as Pdm09 did [3]..

For the past three decades ~~T~~the dominant~~ed~~~~The dominated~~ R type H1 of influenza A changed annually~~ during its outbreak periods~~ [5]~~;~~ by changing its most variable residues,~~:~~ the outermost surface amino acids of the protein structure~~, during three decades~~. In contrast, the inner core region of the protein was conserved~~ative~~; this suggests that the selective pressure is in escaping ~~to the~~ immunity. The new Pdm09 is also changing annually. Coronaviruses have shown few annual changes (Fig. 3B and S6A[5]~~; by changing its most variable residues during three decades, and Pdm09 also changed annually. Coronaviruses have shown few annual changes (Fig. 3b and S6a~~), which might be due to their limited infectibility (HCoV) or ~~to~~ the lack of infected people (MERS-CoV). SCoV2 will face the selective pressure of herd immunity (b) as influenza A ~~virus~~ did. If it escapes this selective pressure, it will remain among humans and spread every few years. Actually, the change in SCoV2 has already begun; they have formed several classes within a~~the~~ short emergence time (Fig. 3C, 6B~~3c, 6b~~). The magnitudes of the PCs~~PC~~ may indicate~~show~~ the migration pathways of the classes. They might mutate within China~~, and~~ transfer~~red~~ to other countries~~,~~ and mutate further (S6 Fig.). These~~The~~ changes may help acclimatisation~~could be acclimation~~ to humans (c); however, they may also relate to ~~the~~ herd immunity (b) and/or lower lethality (d).

Formatted: English (United Kingdom)
Formatted: English (United Kingdom)
Formatted: English (United Kingdom)
Formatted: English (United Kingdom)
Formatted: English (United Kingdom)
Formatted: English (United Kingdom)
Formatted: English (United Kingdom)
Formatted: English (United Kingdom)
Formatted: English (United Kingdom)
Formatted: English (United Kingdom)
Formatted: English (United Kingdom)
Formatted: English (United Kingdom)
Formatted: English (United Kingdom)
Formatted: English (United Kingdom)
Formatted: English (United Kingdom)
Formatted: English (United Kingdom)
Formatted: English (United Kingdom)
Formatted: English (United Kingdom)
Formatted: English (United Kingdom)
Formatted: English (United Kingdom)

~~Fortunately, the lifespan of the classes of coronaviruses should be shorter than that of the influenza virus. The ORF lengths for the influenza virus are within a certain range, but some of the ORFs of coronavirus are quite short, e.g. the envelope protein, which is located in the conserved outermost region of the virus~~ [34] ~~(Fig. 2 and S3).~~ Fortunately, the lifespan of the classes of coronaviruses should be shorter than those~~at~~ of ~~the~~ influenza viruses. The ORF lengths for ~~the~~ influenza viruses are within a ~~certain~~ range of 2.3k-0.9Kb~~k~~ [9] and all ~~the~~ ORFs ~~were~~change annually ~~changed~~ at the same rate~~velocity~~ [5]. ~~However, i~~In contrast, some of the coronavirus ORFs ~~of coronavirus~~ are quite short~~,~~. For example,~~e.g.~~ the envelope protein [35]~~,~~ ~~which~~ is only 260 bases long and located in the conserved region of the ~~virus~~ genome [34] (Fig. 2 and S3). ~~It seems that t~~This protein ~~is~~might be too short to form a variable structure~~.~~, ~~Therefore, this will be~~making it a good target for herd immunity. ~~and t~~These conservative ORFs might be suitable ~~to produce~~ vaccine~~s~~ targets. In contrast, the ~~S~~spike protein tended~~tends~~ to change (S3 Fig.)~~,~~ and may cause antibody-dependent enhancement (ADE), since it covers the surface of the virus. In ADE, the antibody that binds to the virus ~~body~~ may help it~~to~~ enter the target cell through Fc or complement receptors [36]~~,~~ (~~besides~~in addition to the ~~ordinal~~ Ace2~~-~~/Spike protein ~~targeting~~receptors [37]). There is evidence that~~Actually,~~ this occurs with~~could be caused in the case of Spike protein of~~ the SARS-CoV spike protein~~virus~~ [38] and there is concern~~ed~~ ~~for~~about SCoV2 [39]. ~~Any of the~~ Influenza proteins showed annual changes at the same ~~speed~~rate, showing that any of the viral components would be under the same selective pressure [5]. If this is also true in coronaviruses~~coronavirus~~, recognising~~recognizing~~ proteins other than the ~~S~~spike protein will help with understanding the immune mechanisms.

~~It seems that this protein is too short to form a variable structure. Therefore, this will be a good target for herd immunity and these conservative ORFs might be suitable to produce vaccines. In~~

Formatted: English (United Kingdom)

~~contrast, the Spike protein tends to change (S3 Fig.), and may cause antibody-dependent enhancement~~ [40]~~.~~

Many bat coronaviruses seemed to be able to infect humans. The bat and human viruses are similar (Fig. 1)~~.)~~ and there are more variations in bats, of which we have observed only a part~~ we have observed~~. Due to replication errors and RNA editing, a bat may possess several variants of coronavirus [6].~~virus variants~~ [6]~~.~~ Although host-virus specificities are shown in laboratory experiments [28], as ~~both~~ humans and other animals have individual variations, the barrier would be more fragile in reality. Once an infection occurs, the virus will adapt through mutations~~, as for the SARS virus~~ [27]. Long-term accumulation of mutations in intermediate hosts, such as pigs[27]~~. Long-term accumulation of mutations in intermediate hosts, such as swine~~ for influenza viruses, is not essential. These viruses would have a limited infectious character and cause mild symptoms to bats or other hosts; however, they could show an excessive adaptation to humans as SARS- and MERS-CoV~~ did~~. As humans do not have herd immunity to many classes of coronaviruses (~~they do not contain~~other than HCoV), these might produce a new pandemic. If intermediates are required, the~~its~~ main contribution could be the amplification of the inoculum size and contact frequency. Bats and human habitats are different, and a bat may not ~~egest~~produce enough viral particles~~usesviruses~~ to infect different hosts. The size of the donor animal is important, for example,~~e.g.~~ healthcare workers with secondary MERS infections tend to have milder symptoms and a better prognosis [32].[32]~~.~~ This could be caused by differences in inoculum sizes; as camels produce ~~plenty of~~ nasal secretions full of viruses [31, 41], the inoculum size would be larger than that from humans. To prevent the ~~production~~infection of intermediate animals, live animals of different species should not be kept in the same place.

Formatted: English (United Kingdom)

Formatted: English (United Kingdom)

Formatted: English (United Kingdom)

Formatted: English (United Kingdom)

Formatted: English (United Kingdom)

Formatted: English (United Kingdom)

Formatted: English (United Kingdom)

Formatted: English (United Kingdom)

Formatted: English (United Kingdom)

Formatted: English (United Kingdom)

Formatted: English (United Kingdom)

Formatted: English (United Kingdom)

Formatted: English (United Kingdom)

Formatted: English (United Kingdom)

Additionally, identifying the first patients is essential to prevent a human outbreak. Thus, people who have frequently contact with living wildlife should not staylive in a cosmopolitan city.

Formatted: English (United Kingdom)

Formatted: English (United Kingdom)

The conventional classification system separates coronavirus into four major groups, thefrom alpha to the delta [8]; these classes have been found by a clustering method. It is true that the methodologies always find a tree structure for the relationships;, however, they are not suitable forto estimatingestimate the classification of many data. For example, they cannot accurately depict the relationship between distant samples they cannot show distances among otherapart samples (they only show the directly connected ones). Additionally, they dowill not suggest the root of the tree. Furthermore, they depend on many assumptions that cannotwill never be verified. In the case of coronaviruses, the four-groupfour group structure was far from the results we found by PCA; for example,e.g. the categories of Alpha- and Beta-coronavirus actually contained large variations. Additionally, many of the credits for the classification of original sequencing records were misjudged (this was also true foras well as those of the influenza viruses). Estimating the classification by using phylogenicphylogenetic methods has poor reproducibility, and the misjudgmentsmisjudgements would have been caused by such difficulty. Using an objective method is preferable to determine the attributions [4]. One of the advantagesbeauties of PCA is that it can apply a classification to sets of other data, without affecting the established one. This will easefacilitate finding attributions of new samples.

As a source of pandemic viruses, coronaviruses have many candidates that are new to humans: also, thoseand can become a seasonal viruses, withcausing annual mutations annually. Here, the nucleotide sequences of coronaviruses were evaluated by using direct PCA [4]. The variations ofin coronavirus were much smaller than in influenzaInfluenza and differences among hosts wereare also small (Fig. 1). There were several classes; some of them includedinclude human

viruses, but others did~~do~~ not. SARS, MERS, and COVID-19 belonged to ~~such~~ a class that lacked HCoVs. Some of the ORFs in coronavirus are rather conservative, and hence would be ideal targets for vaccines (Fig. 2). HCoVs are more conservative than influenza~~Influenza~~ (Fig. 3A and S2G), but they also show annual changes (Fig. 3B). SARS-CoV-2 has changed during spreading to the world; it will continue ~~the~~ mutating~~ons~~ if it stays as ~~the~~a pandemic virus.

~~The conventional classification system of coronavirus did not coincide with the relationships of the sequence data; e.g. the categories of Alpha- and Beta-coronavirus were too wide. Additionally, many of the credits for classification of original sequencing records were misjudged as well as those of the influenza virus. Using an objective method is preferable to determine the attributions [4].~~

Acknowledgments~~:~~

The author would like to thank Editage (www.editage.com) for English language editing.

Formatted: Font: +Headings (Cambria), 18 pt, English (United Kingdom)

Formatted: Font: +Headings (Cambria), 18 pt

Formatted: English (United States)

Formatted: Font color: Black, English (United States)

Formatted: Normal

Formatted: English (United Kingdom)

References

1. Yuen K-S, Ye ZW, Fung S-Y, Chan C-P, Jin D-Y. SARS-CoV-2 and COVID-19: The most important research questions. Cell & Bioscience. 2020;10(1):40. doi: 10.1186/s13578-020-00404-4.
2. Rota PA, Oberste MS, Monroe SS, Nix WA, Campagnoli R, Icenogle JP, et al. Characterization of a Novel Coronavirus Associated with Severe Acute Respiratory Syndrome. Science. 2003;300(5624):1394-9. doi: 10.1126/science.1085952.
3. WHO. Coronavirus disease (COVID-19) Pandemic 2020 [cited 2020 23 May]. Available from: https://www.who.int/emergencies/diseases/novel-coronavirus-2019.
4. Konishi T, Matsukuma S, Fuji H, Nakamura D, Satou N, Okano K. Principal Component Analysis applied directly to Sequence Matrix. Scientific Reports. 2019;9(1):19297. doi: 10.1038/s41598-019-55253-0.
5. Konishi T. Re-evaluation of the evolution of influenza H1 viruses using direct PCA. Scientific Reports. 2019;9(1):19287. doi: 10.1038/s41598-019-55254-z.
6. Agol VI, Gmyl AP. Emergency Services of Viral RNAs: Repair and Remodeling. Microbiology and molecular biology reviews : MMBR. 2018;82(2):e00067-17. doi: 10.1128/MMBR.00067-17. PubMed PMID: 29540453.
7. Lauring AS, Frydman J, Andino R. The role of mutational robustness in RNA virus evolution. Nature reviews Microbiology. 2013;11(5):327-36. Epub 2013/03/26. doi: 10.1038/nrmicro3003. PubMed PMID: 23524517; PubMed Central PMCID: PMCPMC3981611.

8. Woo PC, Huang Y, Lau SK, Yuen KY. Coronavirus genomics and bioinformatics analysis. Viruses. 2010;2(8):1804-20. Epub 2010/08/01. doi: 10.3390/v2081803. PubMed PMID: 21994708; PubMed Central PMCID: PMCPMC3185738.
9. McCauley JW, Mahy BW. Structure and function of the influenza virus genome. Biochem J. 1983;211(2):281-94. doi: 10.1042/bj2110281. PubMed PMID: 6191756.
10. Konishi T. Principal component analysis for designed experiments. BMC Bioinformatics. 2015;16 Suppl 18:S7. doi: 10.1186/1471-2105-16-S18-S7. PubMed PMID: 26678818; PubMed Central PMCID: PMCPMC4682404.
11. Talbot HKB, Crowe Jr. JE, Edwards KM, Griffin MR, Zhu Y, Weinberg GA, et al. Coronavirus infection and hospitalizations for acute respiratory illness in young children. Journal of Medical Virology. 2009;81(5):853-6. doi: 10.1002/jmv.21443.
12. S-f Z, J-l T, X-b H, X Z, D-m Z, K Z, et al. Epidemiology characteristics of human coronaviruses in patients with respiratory infection symptoms and phylogenetic analysis of HCoV-OC43 during 2010-2015 in Guangzhou. PLoS ONE 2018;13(1): e0191789. doi: https://doi.org/10.1371/journal.pone.0191789.
13. Song Z, Xu Y, Bao L, Zhang L, Yu P, Qu Y, et al. From SARS to MERS, Thrusting Coronaviruses into the Spotlight. Viruses. 2019;11(1):59. doi: 10.3390/v11010059. PubMed PMID: 30646565.
14. WHO. Severe Acute Respiratory Syndrome (SARS) 2020 [cited 2020 23 May]. Available from: https://www.who.int/csr/sars/en/.
15. What Have We Learned About Middle East Respiratory Syndrome Coronavirus Emergence in Humans? A Systematic Literature Review. Vector-Borne and Zoonotic Diseases. 2019;19(3):174-92. doi: 10.1089/vbz.2017.2191. PubMed PMID: 30676269.
16. WHO. Middle East respiratory syndrome coronavirus (MERS-CoV) 2020 [cited 2020 23 May]. Available from: https://www.who.int/emergencies/mers-cov/en/.
17. Wu F, Zhao S, Yu B, Chen Y-M, Wang W, Song Z-G, et al. A new coronavirus associated with human respiratory disease in China. Nature. 2020;579(7798):265-9. doi: 10.1038/s41586-020-2008-3.
18. Zhou P, Yang X-L, Wang X-G, Hu B, Zhang L, Zhang W, et al. A pneumonia outbreak associated with a new coronavirus of probable bat origin. Nature. 2020;579(7798):270-3. doi: 10.1038/s41586-020-2012-7.
19. Faust JS, del Rio C. Assessment of Deaths From COVID-19 and From Seasonal Influenza. JAMA Internal Medicine. 2020;180(8):1045-6. doi: 10.1001/jamainternmed.2020.2306.
20. CDC. Similarities and Differences between Flu and COVID-19 2020. Available from: https://www.cdc.gov/flu/symptoms/flu-vs-covid19.htm.
21. Ogasawara O, Kodama Y, Mashima J, Kosuge T, Fujisawa T. DDBJ Database updates and computational infrastructure enhancement. Nucleic acids research. 2020;48(D1):D45-d50. Epub 2019/11/15. doi: 10.1093/nar/gkz982. PubMed PMID: 31724722; PubMed Central PMCID: PMCPMC7145692.
22. Wright ES. DECIPHER: harnessing local sequence context to improve protein multiple sequence alignment. BMC Bioinformatics. 2015;16:322. Epub 2015/10/09. doi: 10.1186/s12859-015-0749-z. PubMed PMID: 26445311; PubMed Central PMCID: PMCPMC4595117.
23. R Core Team. R: A language and environment for statistical computing. Vienna, Austria: R Foundation for Statistical Computing; 2017.
24. Elbe S, Buckland-Merrett G. Data, disease and diplomacy: GISAID's innovative contribution to global health. Global challenges (Hoboken, NJ). 2017;1(1):33-46. doi: 10.1002/gch2.1018. PubMed PMID: 31565258.
25. The Chinese SARS Molecular Epidemiology Consortium. Molecular Evolution of the SARS Coronavirus During the Course of the SARS Epidemic in China. Science. 2004;303(5664):1666. doi: 10.1126/science.1092002.
26. Guan Y, Zheng BJ, He YQ, Liu XL, Zhuang ZX, Cheung CL, et al. Isolation and Characterization of Viruses Related to the SARS Coronavirus from Animals in Southern China. Science. 2003;302(5643):276-8. doi: 10.1126/science.1087139.
27. Lu H, Zhao Y, Zhang J, Wang Y, Li W, Zhu X, et al. Date of origin of the SARS coronavirus strains. BMC Infectious Diseases. 2004;4(1):3. doi: 10.1186/1471-2334-4-3.
28. Graham RL, Baric RS. Recombination, Reservoirs, and the Modular Spike: Mechanisms of Coronavirus Cross-Species Transmission. Journal of Virology. 2010;84(7):3134-46. doi: 10.1128/jvi.01394-09.
29. Zhou C, Liu S, Song W, Luo S, Meng G, Yang C, et al. Characterization of viral RNA splicing using whole-transcriptome datasets from host species. Scientific reports. 2018;8(1):3273-. doi: 10.1038/s41598-018-21190-7. PubMed PMID: 29459752.
30. Haber JE. DNA Repair: The Search for Homology. BioEssays : news and reviews in molecular, cellular and developmental biology. 2018;40(5):e1700229-e. Epub 2018/03/30. doi: 10.1002/bies.201700229. PubMed PMID: 29603285.

31. Adney DR, Bielefeldt-Ohmann, H., Hartwig, A. E., & Bowen, R. A. . Infection, Replication, and Transmission of Middle East Respiratory Syndrome Coronavirus in Alpacas. Emerging Infectious Diseases. 2016;22(6):1031-7.

32. Dawson P, Malik MR, Parvez F, Morse SS. What Have We Learned About Middle East Respiratory Syndrome Coronavirus Emergence in Humans? A Systematic Literature Review. Vector-Borne and Zoonotic Diseases. 2019;19(3):174-92. doi: 10.1089/vbz.2017.2191. PubMed PMID: 30676269.

33. Bahir I, Fromer M, Prat Y, Linial M. Viral adaptation to host: a proteome-based analysis of codon usage and amino acid preferences. Mol Syst Biol. 2009;5:311-. Epub 2009/10/13. doi: 10.1038/msb.2009.71. PubMed PMID: 19888206.

34. Surya W, Li Y, Torres J. Structural model of the SARS coronavirus E channel in LMPG micelles. Biochimica et Biophysica Acta (BBA) - Biomembranes. 2018;1860(6):1309-17. doi: https://doi.org/10.1016/j.bbamem.2018.02.017.

35. M S, S S. Structural insight into the role of novel SARS-CoV-2 E protein: A potential target for vaccine development and other therapeutic strategies. PLoS ONE 2020;15(8):e0237300. doi: https://doi.org/10.1371/journal.pone.0237300.

36. Takada A, Kawaoka Y. Antibody-dependent enhancement of viral infection: molecular mechanisms and in vivo implications. Reviews in medical virology. 2003;13(6):387-98. Epub 2003/11/20. doi: 10.1002/rmv.405. PubMed PMID: 14625886.

37. Li W, Moore MJ, Vasilieva N, Sui J, Wong SK, Berne MA, et al. Angiotensin-converting enzyme 2 is a functional receptor for the SARS coronavirus. Nature. 2003;426(6965):450-4. doi: 10.1038/nature02145.

38. Yip MS, Cheung CY, Li PH, Bruzzone R, Peiris JSM, Jaume M. Investigation of Antibody-Dependent Enhancement (ADE) of SARS coronavirus infection and its role in pathogenesis of SARS. BMC Proc. 2011;5(Suppl 1):P80-P. PubMed PMID: PMC3019510.

39. Arvin AM, Fink K, Schmid MA, Cathcart A, Spreafico R, Havenar-Daughton C, et al. A perspective on potential antibody-dependent enhancement of SARS-CoV-2. Nature. 2020. doi: 10.1038/s41586-020-2538-8.

40. Lau SKP, Woo PCY, Li KSM, Huang Y, Tsoi H-W, Wong BHL, et al. Severe acute respiratory syndrome coronavirus-like virus in Chinese horseshoe bats. Proceedings of the National Academy of Sciences of the United States of America. 2005;102(39):14040-5. doi: 10.1073/pnas.0506735102.

41. Adney DR, van Doremalen N, Brown VR, Bushmaker T, Scott D, de Wit E, et al. Replication and shedding of MERS-CoV in upper respiratory tract of inoculated dromedary camels. Emerging infectious diseases. 2014;20(12):1999-2005. doi: 10.3201/eid2012.141280. PubMed PMID: 25418529.

~~**Competing interests:** The authors declare no competing interests.~~

Formatted: English (United Kingdom)

~~**Data and materials availability:** Available in the Supporting information.~~

Supporting information

S1 Fig. Classes of coronaviruses at the presented axes of PCs. Blue: human samples. Labels are the same as in Fig. 1~~.~~ c. Classes found in the Sarbecovirus. SARS-CoV and SCoV2 belong~~ed~~ to different groups.

S2 Fig. Subclasses ~~found in the indicated classes~~ of ~~the~~ Influenza A virus. a. H1 hemagglutinin, **b**. H4 PB1, **c**. H5 hemagglutinin, **d**. H7 PB2, **e**. H9 hemagglutinin, **f**. H9 PB1. Values of the mean distance were indicated. The subclass may coincide with the hosts (**d**) but in many cases, ~~a class was occupied by the limited host~~one host species formed a distinct class.

S3 Fig. Levels of PC1-PC5 at each position in the nucleotide sequences. a. Sarbecovirus, **b**. HCoV OC43, **c**. MERS-CoV. **d**. SCoV2. Names of the ORFs are indicated ~~as~~ in Fig. 2.

S4 Fig. Separation of classes in PC1 and 2. Estimated ~~by~~ using indels (**a**) and substitutions (**b**).

S5 Fig. Subclasses of OC43 were separated by the indicated PCs found in the whole genome. Conservative characteristics of the virus and repeated appearance were obvious.

S6 Fig. a Annual changes in the MERS-CoV genome. b Comprehensive data for SARS-CoV-2. Samples found in some European countries showed higher magnitudes of PCs, indicating accumulation of mutations (also see Table S4).

S1 Table. PC for samples of whole genome sequences of coronaviruses. Figure 1 was made from a part of this table.

S2 Table. Differences in spike proteins a. SARS-CoV and bat SARS-like viruses~~.~~ b. SCoV2 and other coronaviruses. Aligned protein sequences were compared. The IDs for the samples are listed.

Formatted: English (United Kingdom)

Formatted: English (United Kingdom)

Formatted: English (United Kingdom)

Formatted: English (United Kingdom)

Formatted: Font: Not Bold, English (United Kingdom)

Formatted: English (United Kingdom)

Formatted: English (United Kingdom)

S3 Table. PC for samples of HCoV OC43 coronaviruses.

S4 Table. PCs of SARS-CoV-2.

S1 data. Aligned sequences for the coronaviruses~~coronavirus~~.

S2 data. Aligned sequences for the OC43.

S3 data. Aligned sequences for SARS-CoV-2.

Formatted: English (United Kingdom)

Formatted: English (United Kingdom)

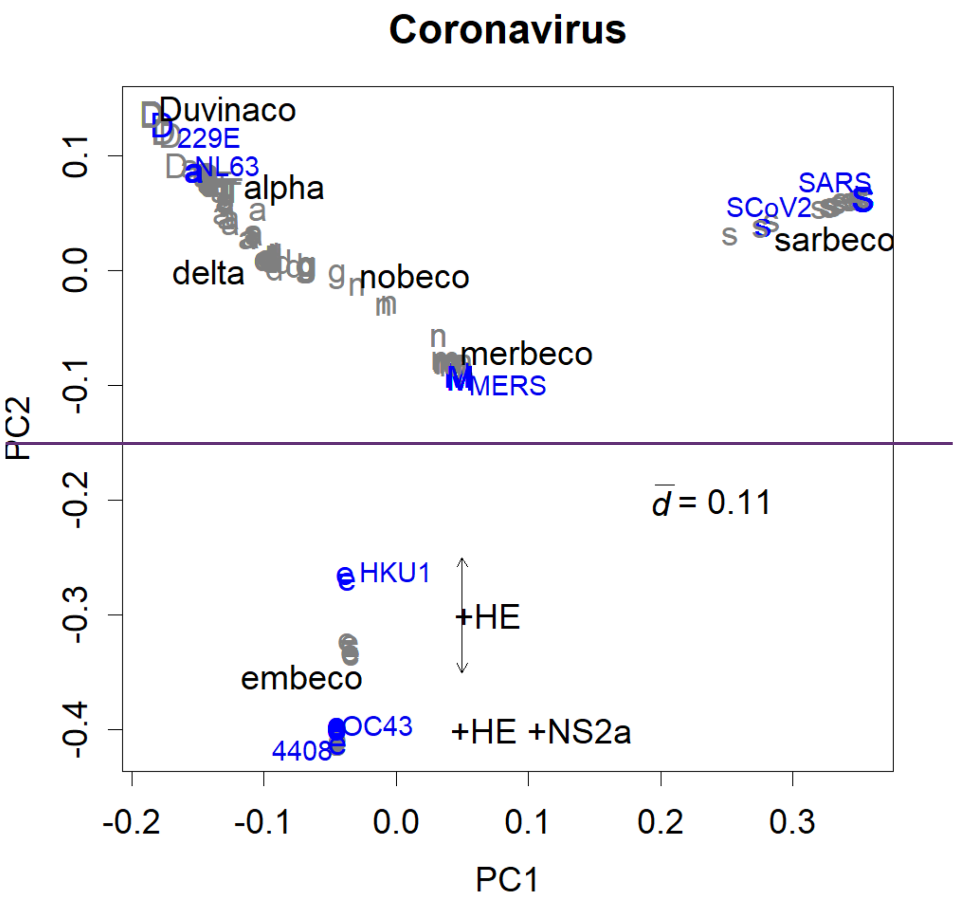

Fig. 1. Classes of coronaviruses separated into PC1 and 2. Blue: human samples; subclasses of HCoV are indicated. a: Alphacoronavirus; d: Delta coronavirus; D: Duvinacovirus; e: Embecovirus; g: Gammacoronavirus; m: Merbecovirus; n: Nobecovirus; s: Sarbecovirus; T: TGEV. A major estimated class, Betacoronavirus, is not shown here, since the class has to cover distinctive classes (Embecovirus, Merbecovirus, Norbecovirus, and Sarbecovirus). It should be

Formatted: English (United Kingdom)

noted that many of the classification credits in the original record were different from this presentation.

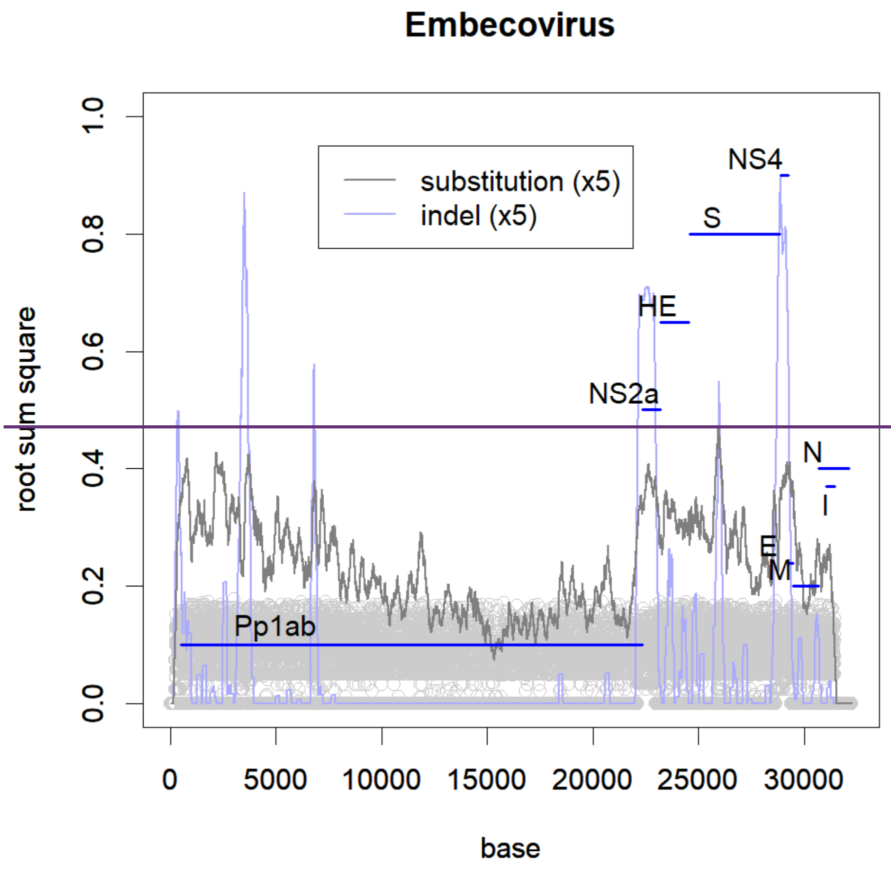

Fig. 2. Frequency of mutations in each position of the genome. The level is estimated by the root sum square of PC1-PC5 (grey circles). The moving averages are represented in a 1:5 scale. The

Formatted: SOMContent

Formatted: English (United Kingdom)

names of the ORFs are indicated. The indel at NS2a reflects that there are subclasses with the ORF and without the ORF.

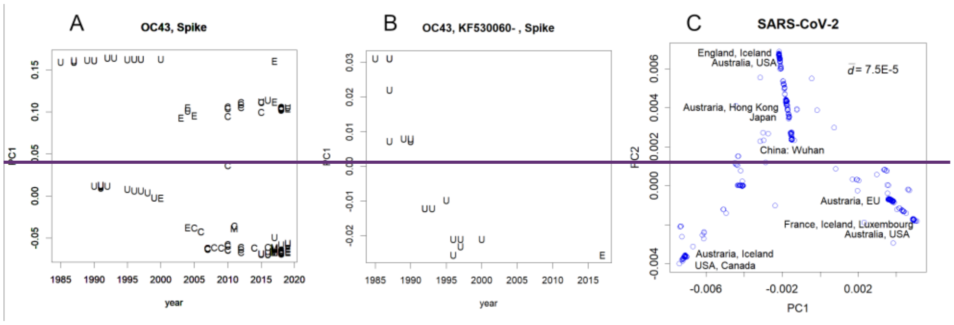

Formatted: English (United Kingdom)

Fig. 3. A. PC of the reported HCoV OC43 spike protein in each year. The letters represent the countries/regions: C- China, E- EU, M- Malaysia, U- USA. The upper-most series of variant were further selected to focus on them in the next panel. **B**, annual changes in the selected variant. Similar one-directional changes found in PC1 were always found in influenza H1N1 human cases [3]. **C**. Classes of SCoV2 separated in PC1 and 2. Data from 2,836 samples are shown. Examples of countries are indicated. The full set of records is presented in Table S4.

Formatted: English (United Kingdom)

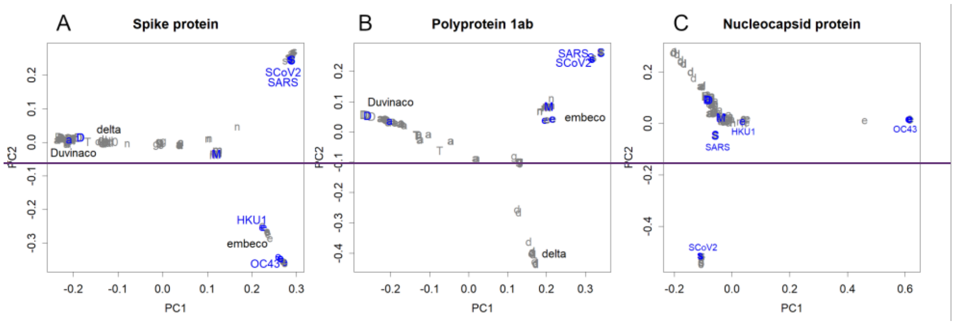

~~**Fig. 4 A.** Classification obtained from the amino acid sequences of the spike protein The relationships between the classes were similar to those estimated from the entire nucleotide sequences (Fig. 1). **B**. Classes found in polyprotein 1ab. The positions of the Deltacoronavirus and the Sarbecovirus were replaced. **C**. Classes found in the nucleocapsid protein. SARS-CoV moved out of the Sarbecovirus class.~~

Formatted: English (United Kingdom)

Formatted: English (United Kingdom)

Formatted: English (United Kingdom)

Formatted: SOMContent
